# Microbiome and Microbiota Within Wineries: A Review

**DOI:** 10.3390/microorganisms13030538

**Published:** 2025-02-27

**Authors:** Cristina Aires, Rita Maioto, António Inês, Albino Alves Dias, Paula Rodrigues, Conceição Egas, Ana Sampaio

**Affiliations:** 1 Centro de Investigação e Tecnologias Agroambientais e Biológicas (CITAB), Universidade de Trás-os-Montes e Alto Douro (UTAD), Quinta de Prados, 5000-801 Vila Real, Portugal; ana.aires@utad.pt (C.A.); ritasantos@utad.pt (R.M.); jdias@utad.pt (A.A.D.); 2 Centro de Química Vila Real (CQ-VR), Universidade de Trás-os-Montes e Alto Douro (UTAD), Quinta de Prados, 5000-801 Vila Real, Portugal; aines@utad.pt; 3 Laboratório Associado Instituto para a Inovação, Capacitação e Sustentabilidade da Produção Agroalimentar (INOV4AGRO), Universidade de Trás-os-Montes e Alto Douro (UTAD), Quinta de Prados, 5000-801 Vila Real, Portugal; 4 Centro de Investigação de Montanha (CIMO), SusTEC, Instituto Politécnico de Bragança, Campus de Santa Apolónia, 5300-253 Bragança, Portugal; prodrigues@ipb.pt; 5 Genoinseq—Next Generation Sequencing Unit, Biocant, BiocantPark, Núcleo 04, Lote 8, 3060-197 Cantanhede, Portugal; conceicao.egas@biocantassociacao.pt; 6 CNC-UC—Center for Neuroscience and Cell Biology, University of Coimbra, UC-Biotech, BiocantPark, Núcleo 04, Lote 8, 3060-197 Cantanhede, Portugal; 7 CIBB—Center for Innovative Biomedicine and Biotechnology, University of Coimbra, UC-Biotech, BiocantPark, Núcleo 04, Lote 8, 3060-197 Cantanhede, Portugal

**Keywords:** bacteria, yeast, fungi, surfaces, vinification, risks, climate change

## Abstract

The main goal of this work is to review the winery’s microbiota, from the grape to the winery’s microbial niches (fermentation tanks, surfaces, air), and their risks to wine and human health. The impact of climate change on the winery microbiome and related challenges are also discussed. Microbial diversity in wineries depends on several factors, such as the grape variety and its ripeness, temperature, relative humidity and the diverse activities of the winemaking process. Winery surfaces and equipment allow the establishment of a microbial community that can impact wine quality, the health of winery workers and visitors and even wine consumers. In the context of climate change, changes in the sugar content, phenolic compounds and the profile of hexoses and amino acids are already evident. These changes interfere with the fermentation microbiota and the quality of the wines, which are more alcoholic and less acidic. Furthermore, periods of drought or heavy rain favor species associated with berry diseases, including some capable of producing mycotoxins or harmful biogenic amines. In order to understand the impact of these changes on microbial communities, the use of various techniques will be discussed, such as flow cytometry, fluorescence in situ hybridization (FISH), quantitative polymerase chain reaction (qPCR) and metagenomic methods.

## 1. Introduction

Humans have an ancient association with vines (*Vitis vinifera* L.) and have produced wine from the dawn of civilization to the present [1]. The oldest evidence of viniculture and winemaking was discovered in South Caucasus, in Gadachrili Gora Georgia [2]. Since then, the production and consumption of wine have been steadily increasing over time all over the world, and they have a huge economic impact annually [3]. The largest wine producers on a global scale are France, Italy, Spain, the United States of America (USA) and Chile, which together represent 63% of total worldwide production [3]. Nowadays, the International Organization of Vine and Wine comprises 50 member countries [4] in five continents, with at least 90,086 wineries [5].

**Figure 1:**
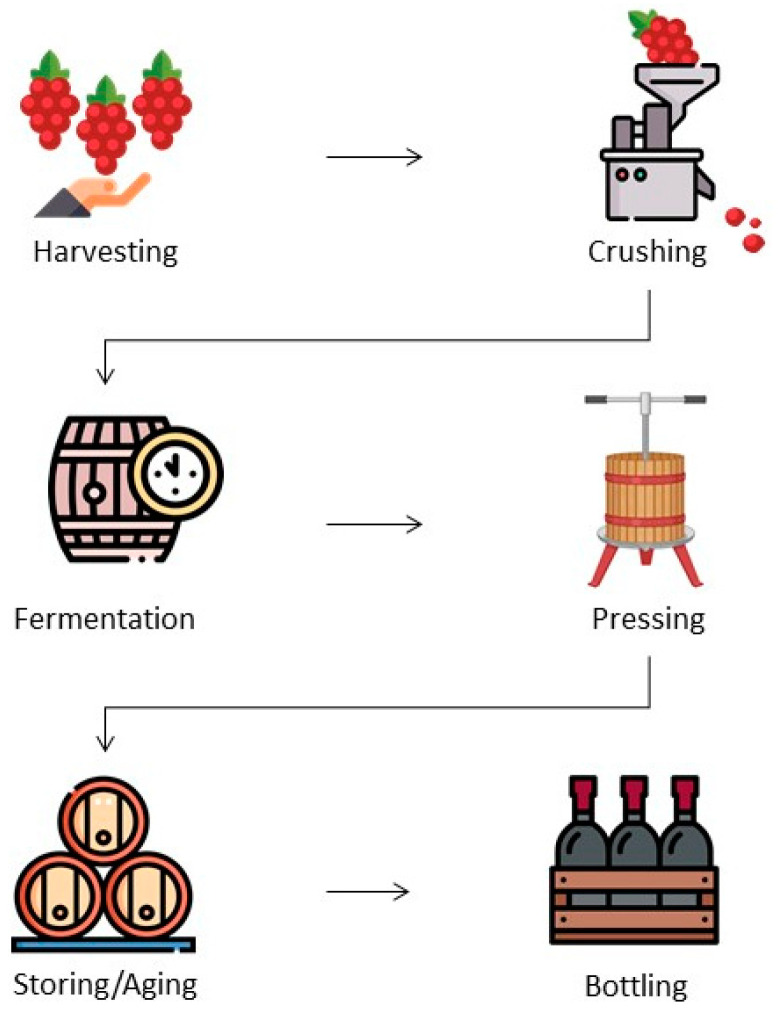
The winemaking process for red wines. In white wines, pressing and clarification occur before fermentation.

Winemaking consists of transforming grape juice into wine. As soon as the grapes arrive at the winery, they are separated from the stalk (destemming), minimizing defects in the wine and preventing the activity of undesirable micro-organisms [6,7]. After destemming, grapes are crushed and, for white wines only, the skins and seeds are removed before fermentation (Figure 1). The fermentation step, naturally driven by the autochthonous microbiota or controlled by commercial strains, is a succession of micro-organisms [6].

After fermentation, the solid residues, such as fruit pulp and yeasts, are deposited at the bottom of the fermentation tanks through gravity; the wine is separated from its sediments and transferred from one container to another (racking) [6]. The suspended compounds that cannot be removed only by decantation, giving the wine a cloudy and opaque appearance, are precipitated by adding coagulants to the wine (clarification). In the clarification step, the wine is also stabilized by removing the tartaric acid [6]. The maturation or aging of wines can be carried out in stainless-steel tanks or in oak barrels [8]. Finally, bottling is the last phase of the winemaking process. Once properly bottled, the wines remain stored in the cellar for as long as necessary.

Wine is a complex matrix that includes components of different chemical nature, such as volatile compounds and phenolics, which are responsible for the aromatic quality of wine. The combination of sensorial and organoleptic characteristics, which constitutes a decisive factor for the consumer to define the quality of the wine, are influenced by the microbial interactions throughout the winemaking process [9]. The concept of “terroir”, initially used as a marketing tool to differentiate wines, is currently used to explain the uniqueness of a wine, and it can refer to the flavor and aroma of the wine, the “wine terroir”, or to the physical, chemical and microbial characteristics of the soil, the “vineyard terroir” [10]. Therefore, the microbial biogeography found in different wine regions is an important determinant of wine terroir [11]. Furthermore, physical factors (e.g., climate and meteorology), soil and biological factors (e.g., grape variety and soil microbiota), as well as viticulture and enological practices, influence the sensory characteristics of a wine in a given region [12].

The main goal of this review is to provide a comprehensive overview of the winery’s microbiota, from the grapevine to the indoor winery (fermentation tanks, surfaces and air). The potential risks associated with the winery microbiota to wine and its consumers, workers and visitors are also presented. The challenges related to climate change and monitoring of microbial communities are also discussed.

## 2. Methodology

In a first literature survey on the study of the microbiome/microbiota within the winery environment, a search was carried out in the Scopus and Web of Knowledge (WOS) databases (July 2024) using 5 keyword filters (“diversity”, “winery”, “yeast”, “mould” and “bacteria”), separated by “or” to encompass the largest number of studies. The publications’ keywords were retrieved from the abstracts, titles and authors’ own keywords. A total of 278 publications (period range 1968–2024) emerged. Duplicate publications and publications related to wastewater treatments, solid waste, landscape, tourism, micro-vinification, fermentation of fruits other than grapes, bioethanol production and enological techniques were excluded.

**Figure 2:**
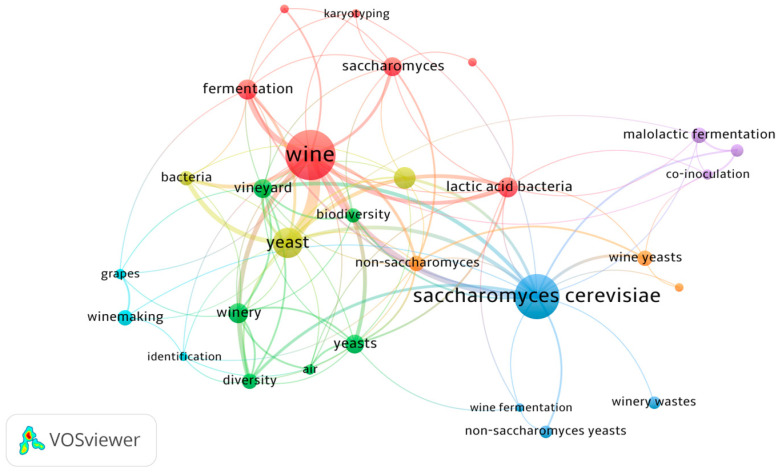
Bibliographic visualization co-occurrence (at least three times) in the references retrieved from the Scopus database (n = 168 publications), obtained using the VOSviewer 1.6.20 tool. The number of co-occurrences of two keywords indicates the number of publications in which both keywords occur in the title, abstract or keyword list. Circles represent nodes, and the larger their size, the more frequent the keyword is. Clusters (indicated by the colors of the circles) are sets of closely related nodes. The lines (thickness and length) indicate the strength of the relationship between the keywords. The font size of keywords is used to represent total link strength (TLS).

A bibliometric visualization map was created from the retrieved publications’ keywords, highlighting the strongest and weakest connections in a general but also intuitive way (Figure 2).

The strongest clusters focus on the study of wine (fermentation, *Saccharomyces*, karyotyping and lactic acid bacteria, LAB) and on the importance of *Saccharomyces cerevisiae*, wine fermentation and its relation to non-*Saccharomyces* yeasts (Figure 2). The third cluster links (bio)diversity in the vineyard, winery, yeasts and air. To a lesser extent, the bibliographic map also highlights the scientific production related to malolactic fermentation (MLF) and the link to the grape microbiome, winemaking and identification.

## 3. Microbiology of Grape Berries, Must and Winery Surfaces

### 3.1. Microbiology of Grape Berries

For 7500 years, humans have sought to understand and control the development of the vine, the maturation of grapes and the alcoholic fermentation to produce wine [13]. From the environment where the vines grow, including soil, topography, climate and weather conditions, to the management practices in the vineyards, the microbes present can potentially change the composition of the wine. The introduction of grapes into the winery and the start of the winemaking process further modify the microbial communities [14]. Despite their technological importance, their origin, prevalence and dissemination routes from the environment to the winery have not yet been fully unveiled.

In vineyards, the most important intrinsic and extrinsic factors that influence wine quality have been identified and include grape-specific differences in secondary microbial metabolite diversity and composition; soil, weather and climate; geological conditions and environmental stress factors; viticulture and the winemaking process [15,16]. The micro-organisms from grapes are of particular importance in spontaneous fermentations. Between the vineyard and the winery, there is a microbial continuum (winegrowing microbiome), and it can be considered that winemaking begins with the grape [17]. Thus, variations of the wine-relevant microbiota in grapes can impact winemaking and wine quality [17,18,19].

The grapes used to make wine have been studied for several decades. The work of Louis Pasteur had already demonstrated the presence of fermentative micro-organisms on the surface of the grape. Grapes have a complex microbial ecology, including filamentous fungi, yeasts and bacteria with different physiological characteristics. The yeast population on sound grapes can go from 10^2^ to 10^5^ CFU/berry, depending on the ripening state [20]. Some species are found only on grapes, such as vine parasitic fungi and environmental bacteria, while others have the ability to survive and grow in wines [19,21]. Also, some parts of the vine, such as roots, leaves and flowers, can share their microbiota with the grape skin [22,23].

The cuticle of healthy berries may present microcracks and soften with berry maturation, increasing nutrient availability and explaining the possible dominance of oxidative or weakly fermentative yeasts, such as the non-*Saccharomyces* species (Table 1). Among them, basidiomycetous (*Cystobasidium Cystofilobasidium*, *Cryptococcus*, *Filobasidium*, *Papiliotrema*, *Rhodotorula*, *Sporobolomyces* and *Vishniacozyma*) and ascomycetous (*Aureobasidium*, *Candida*, *Debaryomyces*, *Hanseniaspora*, *Lachancea*, *Metschnikowia* and *Pichia*) species can be present. With less representation, other genera with fermentative species appear: *Debaryomyces*, *Kloeckera*, *Schizosaccharomyces*, *Saccharomyces*, *Saccharomycodes*, *Torulospora*, *Zygoascus* and *Zygosaccharomyces* [19,24]. These yeasts help in the fermentation process and grow more or less sequentially until the third day of the fermentation process. In the middle and final stages of AF, the more strongly fermenting and ethanol-tolerant species, such as *Saccharomyces cerevisiae*, take over [25,26,27].

According to a study in Italy, the most abundant species found on the surface of the grape was *Aureobasidium pullulans* (59%), and in lower abundances, *Hanseniaspora uvarum*, *Cladosporium ramotenellium*, *Cladosporium cladosporioides*, *Zygosaccharomyces rouxii* and *Starmerella bacillaris* were also found [28]. In Greece, the most abundant species found were *Metschnikowia pulcherrima* and *H. uvarum* (37% and 32%, respectively). The *S. cerevisiae* species was detected at a low abundance (<5%) [29]. Also, in Greece, in northeast Macedonia, the grape carposphere was dominated by Ascomycota (mean 87.9%), followed by Basidiomycota (mean 12.1%). The most abundant fungal taxa belonged to *Aureobasidium* sp., *Cladosporium* sp. and *Alternaria* sp. [30]. Another study spanning nine regions of China showed that, in all of them, the most abundant genus in grapes was *Hanseniaspora* [24,31]. In Croatia, a metagenomic analysis of fresh grape berries from 11 vineyards along the Dalmatia coast showed the dominance of *Aureobasidium*, followed by *Cladosporium*, *Metschnikowia* and *Hanseniaspora*. The fungal community seems to be more influenced by climate conditions and soil type than the bacterial community [24]. Also, yeast species seem to vary along the grape development stages: *A. pullulans* and *Rhodosporidium babjevae* were detected at the early stages; *Pichia anomala* and *H. uvarum* were detected after veraison; and species from *Saccharomyces* and *Candida* were mainly detected on mature grapes [20].

In grapes, the bacterial communities are dominated by *Proteobacteria* [22,30,32] (Table 1). The grape bacterial community included lactic acid bacteria (LAB; *Lactobacillus*, *Oenococcus* and *Pediococcus*) and acetic acid bacteria (AAB; *Acetobacter* and *Gluconacetobacter*), involved in malic acid conversion and wine spoilage, respectively [19,24]. In Long Island, USA, *Proteobacteria* dominated the grape microbiota, followed by *Firmicutes*, *Acidobacteria* and *Bacteroidetes*. The most abundant genera in grapes were *Pseudomonas* (19%), *Sphingomonas* (33%) and *Methylobacterium* [22]. In Lussac St. Emilion, southwest France, in Merlot grapes, the culturable bacteria were dominated by *Pseudomonas*, *Massilia* and *Micrococcus* [32]. In Greece, the bacterial microbiome of grapes was mainly composed of *Proteobacteria* (mean 57.7%), *Actinobacteriota* (mean 15.8%) and *Firmicutes* (mean 15%), with *Pseudomonas*, *Sphingomonadaceae*, *Massilia* and *Methylobacterium* accounting for 35.5% in all samples [30].

The microbial diversity in the vine and wine ecosystems described in the various works differs with the grape variety, vintage, geographical location of the vineyard, intra-vine interactions, sampling methods and identification techniques, making comparisons between wine regions, varieties and vintages very difficult [30,33]. Although the importance of grape quality in wine quality is recognized, overall, the influence of the grape microbiota on the regional characteristics of wines, a phenomenon known as “microbial terroir”, is poorly understood or undefined [9]. As an example, distinct genotypes of *S. cerevisiae* from different regions of New Zealand have a different impact on the wine chemical profile [34]. The concept of microbial terroir can be extended to non-*Saccharomyces* wine species, such as *Lachancea thermotolerans* vineyard strains in Greece, and *H. uvarum* genotypes from different regions of France and South Africa [35,36].

Vine and grapes can be affected by diseases caused by viruses, bacteria, fungi and oomycetes. In grapes, the most common diseases are downy mildew (*Plasmopara viticola*), powdery mildew (*Erysiphe necator*, syn. *Uncinula necator*), gray rot (*Botrytis cinerea*), black rot (*Guignardia bidwellii*) and sour rot (several yeasts and bacteria involved), which are mainly prevented by the application of phytochemicals. In addition, grapes may also contain other fungi (e.g., *Alternaria*, *Cladosporium* spp., *Aspergillus* spp., *Penicillium* spp.) responsible for various grape rots or mycotoxin production. However, these fungi cannot grow in must and wine, and their effect on wine quality is due to damaged grapes [19,37,38]. *Aspergillus carbonarius* and *Aspergillus niger* are the dominant members of the pathobiome of grapes [39]. They are opportunistic fungi that develop mainly in berries damaged during ripening [40] and are also the main producers of mycotoxins in grapes (in particular ochratoxin A). The “black” *Aspergillus* (section *Nigri*) causes the *Aspergillus* bunch rot in grapes, which makes them soft and dull and covered with brown or black spores. This defect is impacted by agronomic practices, excessive irrigation, grape berry damage due to birds, insects or infection by other fungi, or caused by periods of rain that cause the berry to burst [41,42].

**Table 1:** Microbiota/microbiome studies (2005–2025) on/in grape berries ordered by publication date.

Sample	Methods	Sampling	Fungi Taxa	Bacteria Taxa	Location, Country	Reference
Grapes from Merlot, Cabernet Sauvignon and Cabernet Franc	Cultural methods and PCR-RFLP-ITS (fungi), PCR-DGGE (bacteria)	Grape from 3 vineyards at different stages (berry set for harvest)	*A. pullulans*, *Yarrowia lipolytica*, *C. boidinii*, *Candida fructus*, *Candida intermedia*, *Candida membranifaciens*, *Candida stellata*, *Lipomyces spencermartinsiae*, *Met*. *pulcherrima*, *Debaryomyces hansenii*, *Issatchenkia orientalis*, *Issatchenkia terricola*, *Kluyveromyces lactis*, *P*. *anomala*, *Pichia fermentans*, *Saccharomyces boulardii*, *S*. *cerevisiae*, *H*. *guillermondii*, *H*. *opuntiae*, *H*. *uvarum*, *Bulleromyces albus*, *Cr*. *albidus*, *Cr*. *flavescens*, *Papiliotrema laurentii*, *Rhodosporidium babjevae*, *Rh*. *glutinis*, *Rh*. *graminis*, *Rh*. *mucilaginosa*, *Sporidiobolus pararoseus*, *S*. *salmonicolor*, *Sporobolomyces roseus*	*Enterobacter*, *Serratia*, *Gluconobacter oxydans*, *O. oeni*, *Pediococcus parvulus*, *Burkholderia vietnamiensis*	Not mentioned, France	[20]
Merlot grapes from two vineyards	Cultural methods and T-RFLP	Beginning of grape ripening, healthy and undamaged grapes		*Pseudomonas*, *Massilia*, *Micrococcus Curtobacterium* *Brevibacterium* *Enterobacter, Burkholderia*	Lussac St. Emilion, France	[32]
Merlot grapes from five vineyards	Illumina (NGS)	During harvesting		*Sphingomonas,* *Pseudomonas, Methylobacterium*	Long Island, USA	[22]
Sauvignon blanc grapes	Pyrosequencing (NGS)	During harvesting	*Cladosporium*, *Davidiella* *Columnosphaeria*, *Botryotinia* *Torulaspora*, *Saccharomyces* *Hanseniaspora*, *Pleosporales* *Trichocomaceae*		Six wine regions (36 vineyards), New Zealand	[23]
Grapes from Peloponnese peninsula, Santorini, Crete (33 vineyards)	Culture followed by PCR-RFLP	Grapes from 2–9 sampling plots per vineyard	*A. pullulans*, *C. diversa*, *C. glabrata*, *C. membranifaciens*, *C. tropicalis*, *Cr. diffluens*, *H. uvarum*, *H. guilliermondii*, *H. opuntiae*, *Hyphopichia pseudoburtonii*, *L. thermotolerans*, *Meyerozyma caribbica*, *Met. pulcherrima*, *Papiliotrema laurentii*, *Pichia anomala*, *S. cerevisiae*, *Starmerella bacillaris*, *Torulaspora delbrueckii*		Peloponnese peninsula, Santorini and Crete islands, Greece	[29]
Grape varieties Faith and Gratitude	Illumina (NGS)	Grape berries 7 days before harvest	*Meyerozyma*, *Candida*, *Cladosporium*, Saccharomycetales, *Filobasidium*, *Papiliotrema*, *Zygoascus*, *Hanseniaspora*, *Hannaella*, *Didymella*, *Dissoconium* Less abundant: *A. pullulans*, *Metschnikowia*, *Pichia*, *Rhodotorula*, *Cryptococcus*		Not mentioned, USA	[43]
Grapes from Cabernet Sauvignon, Marselan, Merlot, Chardonnay, Petit Manseng, Petit Verdott, Cabernet Franc, Beimei, Beihong, Syrah, Vidal	Cultural methods; molecular identification	Grape berries near ripening/harvest (11 varieties, 9 wine regions, 3 vineyards, 2 consecutive years)	*H. uvarum*, *H. opuntiae*, *H. occidentalis*, *H. vineae*, *R. paludigena*, *R. nothofagi*, *C. glabrata*, *C. apicola*, *C. xestobii*, *C. silosis*, *M. caribbica*, *M. guilliermondii*, *Met. pulcherrima*, *Met. chrysoperlae*, *K. exigua*, *K. hellenicae*, *Cr. flavescens*, *Issatchenkia terricola*, *S. bacillaris*, *T. delbrueckii*, *K. marxianus*, *Z. bailii*, *Clavispora lusitaniae*, *A. pullulans*, *Wickerhamomyces anomalus*, *Cyberlindnera fabianii*		Several regions, China	[31]
Grapes from two vineyards (organic versus conventional management)	Culture followed by molecular identification of the isolates	Grape berries 6/8 days before harvest	*A. pullulans*, *Cladosporium ramotenellium*, *C. cladosporoides*, *Naganishia globosa*, *F. magnum*, *H. uvarum*, *Zygosaccharomyces rouxii*, *S. bacillaris*, *Paraconiothyrium* sp.		Valpolicella DOC wine region, Italy	[28]
Grapes of Maraština cultivar (11 vineyards)	Illumina (NGS)	Mature, healthy and undamaged grapes	*Alternaria*, *Aureobasidium* *Cladosporium*, *Filobasidium* *Hanseniaspora*, *Metschnikowia* *Quambalaria* (<1%): *Botrytis*, *Buckleyzyma*, *Cryptococcus*, *Cystobasidium*, *Didymella*, *Eremothecium*, *Hyphopichia*, *Penicillium*, *Pichia*, *Plenodomus*, *Sporobolomyces*		Dalmatian coast, Croatia	[24]
Grapes of red wine cultivars from 7 vineyards	Illumina (NGS)	During harvesting	*Alternaria*, *Aureobasodium*, *Cladosporium*, *Curvularia*, *Filobasidium*, *Pseudopithomyces* *Vishniacozyma*	*Pseudomonas Sphingomonadaceae Massilia Methylobacterium*	Northeast Macedonia Region, Greece	[30]

PCR-RFLP-ITS—Polymerase chain reaction-restriction fragment length polymorphism of the internal transcribed spacer; PCR-DGGE—PCR-denaturing gradient gel electrophoresis; T-RFLP—Terminal restriction fragment length polymorphism; PCR-RFLP—Polymerase chain reaction-restriction fragment length polymorphism; NGS—Next-generation sequencing.

### 3.2. Microbiology of Must

Wine is the result of a complex biochemical process, which begins at the harvest and extends to alcoholic (AF) and malolactic (MLF) fermentations, aging and bottling [44]. Winemaking involves a great diversity of micro-organisms with different roles in the process. The microbial consortium of wine includes mostly yeasts, LAB and AAB with different implications for wine quality [45]. From a microbiological point of view, AF and MLF are the stages of greatest interest, with a heterogeneous microbiota of yeasts, bacteria and filamentous fungi, with considerable possible interaction between them [6]. Although the entire microbiota contributes to the chemistry of wine, yeasts play a preponderant role, as they promote AF (*Saccharomyces* and non-*Saccharomyces* species), a biochemical process in which grape sugars are converted into ethanol and carbon dioxide, also producing many additional compounds (e.g., esters and higher alcohols).

Grapes and musts harbor a complex microbiome, which plays a crucial role in wine fermentation and impacts wine flavor and, subsequently, its final quality and value. Unveiling the microbiome and its dynamics, and understanding the ecological factors that explain such biodiversity, has been a challenge to enology [46]. The yeasts present in the musts originate from the grape bunches and leaves that enter the winery, as well as from the environment of the winery itself. In the early stages of fermentation, the non-*Saccharomyces* species, especially *Hanseniaspora*, *Candida*, *Pichia* and *Metschnikowia*, dominate over the *Saccharomyces* species and are the promoters of spontaneous alcoholic fermentation. As long as alcoholic fermentation takes place, these yeasts are repressed by *S. cerevisiae*, which dominates the intermediate and final stages of the process [44,47,48]. However, non-*Saccharomyces* species can be present during the entire fermentation process [49], contributing to the wines’ aromatic complexity and, subsequently, to their authenticity and regionality [50].

**Figure 3:**
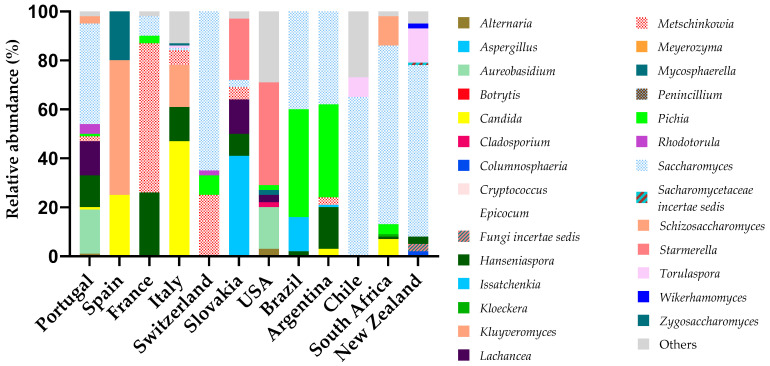
Relative abundance of fungi, at the genus level, during must fermentation in different countries. The relative abundances observed are the result of the artificial average (in percentage) of various musts at different days/stages of fermentation relative to the following studies: Portugal [46], Spain [47], France [51], Italy [52], Switzerland [49], Slovakia [53], USA [54], Brazil [55], Argentina [56], Chile [57], South Africa [58], New Zealand [23].

Figure 3 evidences the fungal diversity among wine production sites, which can vary depending on the region, fermentative stages and vintages.

In Figure 3, the genus that stands out as the most expressive is *Saccharomyces*, present in almost all regions, and its relative abundance is mostly dependent on the fermentative stages. For example, in Slovakia, the genera *Issatchenkia* and *Starmella* stand out, most probably because the data correspond to the must in its initial stage [55]. Contrarily, in a three-year study in two geographic areas of Spain, throughout must fermentation (beginning, middle and final stages), *Saccharomyces* was the most frequent genus in the middle and in the final fermentative stages [59].

Yeasts of *Hanseniaspora* (anamorph *Kloeckera*), *Pichia*, *Candida*, *Metschnikowia*, *Kluyveromyces* and *Saccharomyces* were present in grape musts from various regions (n = 6) of Portugal [46]. It is possible to note the similarity in the diversity profile of yeasts, but also their approximate percentage of expression, found in Brazil and Argentina, two geographically close countries. Occasionally, species from other genera, such as *Zygosaccharomyces*, *Saccharomycodes*, *Torulaspora*, *Dekkera* and *Schizosaccharomyces*, may be present in Portugal [46] and Italy [52].

The frequency of non-*Saccharomyces* taxa varied greatly among the terroirs (Figure 3). *Hanseniaspora* and *Candida* are the main detectable genera in must at the beginning of alcoholic fermentation [44]. *Metschnikowia* species show β-glucosidase activity, which hydrolyzes glucoside-bonding aromatic precursors and releases aromatic compounds, such as terpenes [31].

Fermentative species also include technologically relevant spoilage yeasts due to their ability to deteriorate wines either by producing an unpleasant flavor or through the formation of sediments and turbidity [19]. The transformation of hydroxycinnamic acids, *p*-coumaric and ferulic acids, into volatile phenols is associated with the activity of yeasts of the genus *Brettanomyces/Dekkera* [60].

Regarding the bacterial community of musts, the literature is not as extensive as for yeasts. Most of the studies targeted bacteria responsible for MLF, a process carried out by LAB that are present throughout winemaking and remain in the winery equipment after MLF [6,61]. These bacteria are considered to originate from the grape, but they have also been isolated from vine leaves, grapes, winery equipment and barrels. Lactic acid bacteria are found in small numbers during the initial stages of alcoholic fermentation and belong mainly to the genera *Lactobacillus* and *Pediococcus*. However, as alcoholic fermentation progresses, only bacteria that are able to resist alcohol, such as *Oenococcus oeni*, can survive, thus becoming the main agents in MLF [61]. According to González-Arenzana and collaborators [62], in four Spanish wineries, *O. oeni* dominates (78%) in the final fermentation stages. *Leuconostoc mesenteroides* and *Lactobacillus* sp. comprise the remaining malolactic microbiota present.

In Pennsylvania, a study on the evolution of bacteria between the first and tenth days of fermentation shows that *O. oeni* increases by 46% on average from the fifth day onwards. Other bacterial taxa were found at this stage: *Sphingomonas* (10%), *Enterobacteriaceae* (10%), *Methylobacterium* (9%), *Pseudomonas* (10%), *Lactobacillus* (4%), *Komagataeibacter* (6%) and *Gluconobacter* (4%) [54]. In musts from Portuguese wine regions, *Enterobacteriaceae* was the most significant family, with approximately 68% incidence. Other families found, although with a contribution to the microbiome of less than 10%, were *Halomonadaceae*, *Sphingomonadaceae*, *Oxalobacteraceae, Microbacteriaceae*, *Acetobacteriaceae*, *Xanthomonadaceae*, *Pseudomonadaceae* and *Comamonadaceae* [46].

Acetic acid bacteria are ubiquitous, strict aerobic bacteria that occur in sugary, alcoholic and acidic niches. For the most part, AAB belonging to *Acetobacter* and *Gluconobacter* genera represent spoilage micro-organisms during wine production, mainly because they oxidize ethyl alcohol into acetic acid [63]. Other species detected were *Erwinia gerundensis*, *Acinetobacter* sp., *Escherichia coli*, *Gluconobacter oxydans* H24, *Oryzias melastigma*, *Pantoea agglomerans*, *Pseudomonas* sp. and *Telluria mixta* [53].

A study of the microbial community in two wines over 2 years showed that, on average, *O. oeni* was the predominant species (81%). *Enterococcus faecium*, *Leuconostoc mesenteroides*, *Pedicoccus acidilacticipertainam* and bacteria belonging to the genus *Lactobacillus* (e.g., *Lactobacillus fermentum*, *Lactobacillus casei* and *Lactobacillus plantarum*) represented the remaining percentage [64].

### 3.3. Microbiology of Winery Surfaces

During the transformation of grapes into wine, fermentative processes are exposed to a large surface area within wineries, which can serve as important reservoirs for the bidirectional transfer of microbes between fermentations. Under normal cleaning conditions, wine cellar surfaces have their own microbial community. Surface microbial communities depend on the production context at each location, shaped by technological practices, processing stage and season. It is during the harvest phase that a greater proliferation of organisms associated with the grape and fermentation is observed, populating most of the winery surfaces [65].

The literature on the microbiota of winery surfaces, that is, equipment, floors and other surfaces, is very limited. The knowledge of the bacterial community was studied in a winery in California at the family taxonomic level [65]. In this research, samples were obtained at three different times (before, during and after harvesting), and the microbial load was higher during the harvest period. Also, there were areas with specific microbial niches depending on the season; for example, in equipment where grapes were processed, there tended to be a seasonal community.

Some of the most abundant bacteria in the equipment (e.g., crusher, press, fermentor, floor, pump) belonged to *Burkholderiales*, *Pseudomonadales*, *Actinomycetales*, *Alteromonadales*, *Bacillales*, *Clostridiales*, *Caulobacterales* and *Rhizobiales*. In particular, the barrels’ surfaces were dominated by *Pseudomonas* (*Pseudomonadales*) and *Shewanella* (*Alteromonadales*) [65]. The taxa *Sphingomonas* (*Sphingomonadales*) and *Micrococcaceae* (*Micrococcales*) represented around 20% and 25%, respectively, of the microbial diversity in an Australian winery (in Adelaide), followed by *Brevundimonas* (*Caulobacterales*). Compared to wineries in the USA, the diversity of bacterial genera in Australia is low [66].

**Figure 4:**
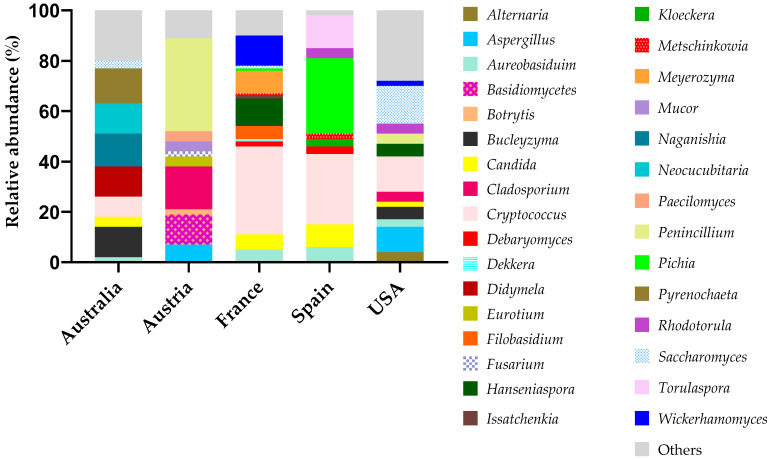
Relative abundance of most fungi, at the genus level, found on winery surfaces in different countries: Austria—on barrel surfaces [67]; Australia—on winery concrete floor [66]; France—sampling average over 5 times in equipment [68]; Spain—winery surfaces and equipment [47]; and USA—winery surfaces [68].

The diversity of yeasts varies with the geographical location and the type of surface analyzed. Figure 4 shows that *Cryptococcus* and *Candida* species were found in almost all studies analyzed [47,66,67,68]. In a controlled fermentation in France, the *Cryptococcus* genus achieved as much as 70% of the fungal population [33]. In addition to *Cryptococcus* (*Cr. difluens* and *Cr. laurentii*), other genera mainly contribute to the surfaces and equipment microbiome, as is the case for *Candida* (*C. sorbosa*, *C. vinaria*, *C. intermedia*, *C. zeylanoides* and *C. wickerhamii*) and *Pichia* (*P. anomala* and *P. membranifaciens*) in Spain [47]. *Torulaspora delbrueckii* and *Zygosaccharomyces* (*Z. veronae* and *Z. bailii*) were only found in Spain [47,69]. Generally, these types of equipment involve porous surfaces that are difficult to clean. Embedded in the nutritive medium of grape juice, these surfaces become very promising sites for microbial adsorption and biofilm production, potentially leading to continued dispersion of microbes in successive batches of wine [65]. Some genera, such as *Alternaria* sp. and *Buckleyzyma* sp., were also reported on floors, walls and equipment, achieving 70% and 3% on walls, respectively [68].

In 1984, Rosini demonstrated that, in the months following winemaking, *Saccharomyces* species, mainly *S. cerevisiae*, colonized the surfaces of the equipment and the winery itself. *Saccharomyces cerevisiae* was considered the main colonizer species of winery surfaces [70]. However, the relative abundance of fungi on surfaces (Figure 4) shows that *Saccharomyces* spp. was neither the most expressive genus nor detected on winery surfaces in Spain and France. *Saccharomyces* is typically associated with fermentative states, as discussed in point 2.2.

The *Dekkera* genus was detected on equipment, floors and walls in a wine cellar in France [68]. This genus, and its anamorphic form *Brettanomyces*, is associated with the transformation of hydroxycinnamic acids into volatile phenols that translate into unpleasant aromas described as “horse sweat”, “animal”, “leathery” and “medicinal”, which affect the quality of the wine [71].

In Austria, samples from walls and wooden barrels with visible mold growth showed *Penicillium* as the predominant genus, with a relative abundance of 37% [67]. Other molds, such as *Cladosporium* sp., *Fusarium* sp., *Mucor* sp., *Trichoderma* sp., *Aspergillus versicolor* and *Alternaria alternata*, were also reported. In swab samples from barrels, *Cladosporium* sp., *Fusarium* sp., *Mucor* sp., *Rhizopus* sp., *Epicoccum nigrum*, *Eurotium herbariorum* and non-identified Basidiomycota were also detected. The surfaces studied were wooden barrels; therefore, one would expect to find these micro-organisms. Some molds, such as *Aspergillus fumigatus*, decreased with higher indoor temperature and relative humidity but increased in cellars built of bricks. Also, *Zasmidium cellare* (syn. *Cladosporium cellare*), the distillery or cellar mold, was found in five brick cellar walls. Contrarily, *A. alternata* increased in wineries built of concrete [67]. Other species found on barrel surfaces from industrial and traditional wineries in Italy were *Aspergillus nidulans*, *A. niger*, *Cladosporium cladosporioides*, *Penicillium decumbens*, *Penicillium expansum*, *Penicillium glabrum*, *Penicillium janthinellum*, *Penicillium verrucosum*, *Trichoderma viride* and *Ulocladium botrytis*. Some of these were also detected on walls of both cellars [72]. The walls of modern cellars seem to be resistant to mold establishment and growth [73]. In Hungary, dark and green colonies of *Zasmidium cellare* covered the walls of all subterranean and old cellars but not those of modern constructions (10 years old). Also, *Bjerkandera adusta*, a Basidiomycota, and *Aspergillus spelunceus* frequently colonize some surfaces in subterranean cellars [73].

Several factors can contribute to the number and composition of microbial populations on contact surfaces. Depending on their composition and sanitary conditions, ingredients, raw materials and process water entering the food processing facility can introduce new microbial populations that may differ from batch to batch. Additionally, microbial sources can include contaminated air, mishandling of industrial waste and operators in the food industry. When conditions are conducive to microbial growth, they can also change over time in response to factors such as the presence of organic waste, variations in cleaning and disinfecting practices, changes in temperature (e.g., during different seasons) and other factors [74].

### 3.4. Air Microbiology

Air microbiology plays a crucial role in various industries, including wineries. Indoor relative humidity (RH), organic matter contamination, the presence of stagnant water and temperature stability provide the necessary conditions for airborne micro-organisms [75]. Microbial air quality can have an impact on the quality of the final product and on the health of workers and visitors. Nevertheless, and despite the growing interest in studying the indoor air microbiota in food industries, in wineries, this interest has been decreasing. This disinterest can result from several challenges associated with airborne micro-organisms. The absence of a standardized air sampling method is the main obstacle in the comparison and reliability of results between studies [76]. Another obstacle is the influence of the type of air collectors on the density and diversity of micro-organisms. In Poland, fungal air densities and diversity depended on the air sampler: more *Cladosporium* spp. were collected with the MAS-100 sampler; more *Fusarium* and *Aspergillus* were collected with the Air IDEAL; and more *Penicillium* spp. were collected with the SAS super 100 [77].

Most studies of air microbiota in wineries have used air impaction/impingement methods, and only one has used the sedimentation method (Table 2). The impaction/impingement method consists of a known volume of air impacting/impinging on the surface of a culture medium, generally selective for a microbial group. The sedimentation method relies on gravity for microbial particles to settle on solid plate media [78], on the types of methods for indoor micro-organisms and their advantages and limitations.

Both indoor fungi and bacteria depend not only on the outside air; they are also affected by human activities, especially in confined environments. From the limited number of studies into the microbiology of air in wineries, it is clear that the microbial presence in winery air is directly related to the winemaking processes that are taking place in the winery [75]. After these activities, the particle numbers of four different aerodynamic size classes (0.3–0.5 µm, 0.5–1 µm, 1–5 µm and >5 µm) and compositions changed during grape handling. The number of large particles (1–5 µm and >5 µm) increased 120-fold and 150-fold, respectively, corresponding to the sizes of bacteria, yeast cells or fungal spores [79]. During stemming and crushing, maximum values for bacteria (485,000 CFU/m^3^) and fungi (146,000 CFU/m^3^) were achieved, particularly when the grapes were dirty or rotten. These micro-organisms can be aerosolized, increasing the indoor microbial loads [79].

**Table 2:** Micro-organisms in winery air: sampling methodology, media used and incubation conditions from works cited (2001–2022), ordered by increasing publication date.

Sampling Method	Media	Incubation	Reference
One-stage SAS Compact sampler, 1.2 m above ground (60 L/sample)	MEA, MESA	8–15 days (22 or 37 °C)	[80]
SKC BioSampler system ^1^, 250 L/sample	BSE	40–48 h (30 °C)	[81]
MAS-100 Eco air sampler (≤100 L per sample)	TSA, MCA, META	TSA: 3 days (30 °C) MCA: 3 days (33 °C) META: 4 days (27 °C)	[79]
MAS-100 Eco air sampler, 1.5 m above ground (20, 50, 100 L air)	MEA, CASO-agar, DG18	6 days (RT or 28 °C)	[82]
Air IDEAL 3P air sampler, 1 m above ground (20–100 L)	CGA, CZA, MRS-agar	48 h–7 days (25 °C), 10 days (30 °C, anaerobiosis)	[75]
Air IDEAL 3P air sampler, 1 m above ground (1500 L/sample)	MRS-agar with 50 mg/L of pimaricin	10 days (30 °C)	[61]
Six-stage Andersen-Cascade impactor, cut-off size (d_50_), 1.5 m above ground (56.6 L/sample)	MEA, DG18	7 days (25 °C)	[67]
Air IDEAL 3P air sampler, 1 m above ground (100 L/sample)	CZA	7 days (20 °C)	[83]
Air IDEAL 3P air sampler (500 or 1500 L/sample)	CGA, MR-agar (enriched TSB)	3 days (25 °C)	[84]
Air IDEAL 3P air sampler, 1 m above ground (500 or 1500 L/sample)	CGA, MR-agar (enriched TSB)	3 days (25 °C)	[85]
AirPort MD8 air sampler, 1 m above ground (LAB—1000 L; yeast—250 L per sample)	MRS, CGA	LAB: 5 days (30 °C) Yeast: 2 days (25 °C)	[64]
Air Ideal air sampler (100–250 L)	TSA, TSA-HN ^2^	3 days (37 °C) with or without CO_2_	[86]
One-stage volumetric sieve sampler (SAS IAQ)	PDA, MEA, RBA (with chloramphenicol)	3–8/15 days (25 °C)	[73]
Koch sedimentation method, 0.6 m above ground or ceiling level, 4 h exposure	SDAC (0.5 g/L)	10 days (25 °C)	[87]

MEA—Malt extract agar; MESA—MEA with 20% sucrose; BSE—*Brettanomyces* selective agar; TSA and CASO-agar—Tryptone soya agar; MCA—MacConkey agar; META—MEA with tetracycline; DG18—Dichloran glycerol agar; CGA—Chloramphenicol glucose agar; CZA—Czapek agar; MRS—De Man–Rogosa–Sharpe agar; TSB—Tryptic soy broth; LAB—Lactic acid bacteria; TSA—Tryptic soy agar; PDA—Potato dextrose agar; RBA—Rose Bengal agar; SDAC—Sabouraud dextrose agar with chloramphenicol. ^1^ equipped with a Vac-U-Go sonic sampler. ^2^ TSA-HN, enhanced with hemin, NADH.

Also, micro-organisms can spread via indoor air, contaminating the exposed surfaces. *Saccharomyces cerevisiae* and *O. oeni* isolates from air were clones of the isolates from the fermentation vats [61,64,75].

The RH and temperature are key factors for indoor microbiology, especially for fungi. The range of these parameters can be wide (e.g., Table 3) for both RH (39–93%) and temperature (9.0–23.5 °C). The mean of microbial CFU/m^3^ in the air of wine cellars differed (*p* < 0.05) with the type and age of the building, the type of air conditioning systems, the outdoor air temperature, the RH, the dew point and visible mold colonization in surfaces [67,82]. The air in old traditional semi-subterranean cellars with limestone walls and hard-packed sod floors had a higher concentration of fungal spores (*p* < 0.05) than modern constructions with concrete walls and floors [80]. The indoor RH has an influence on large visible mold growth, contrary to the indoor temperature: in wineries with RH > 72%, both the mold areas on the walls and the CFU/m^3^ in the air increased [67]. On the contrary, mold levels in a Logroño (Spain) winery were not influenced by either the winemaking activity or the sampling date, but they were influenced by the location and floor level. The results obtained by Ocón and collaborators suggest the existence of a stable population of mold in the winery’s indoors, regardless of the season and of the ongoing activity [83].

Also, the indoor air fungi densities varied with the methods used in viticulture and in vinification. Where winegrowers and wine producers used natural methods, such as little or no pesticides, winemaking without SO_2_ and no wine filtration, a high number of airborne microbial loads was observed [80].

Interestingly, the density of indoor airborne xerophilic fungi in wineries without air conditioning or older buildings was higher than in those with conditioning systems and outdoors [67,80]. This type of fungi can be 10× higher indoors than outdoors, depending also on the build materials used [67].

Based on Table 2, Gram-positive bacteria dominate the winery indoor air (500–485,000 CFU or MPN/m^3^), followed by mesophilic (35–146,000 CFU or MPN/m^3^) and xerophilic molds (98–14,000 CFU/MPN/m^3^), yeasts (0–2300 CFU or MPN/m^3^) and Gram-negative bacteria (0–180 CFU or MPN/m^3^). Specific groups linked to the winemaking process, such as LAB and *Brettanomyces*, are also present in the air, and their presence depends on the phase of winemaking—respectively, MLF and bottling: LAB 0–1800 CFU/m^3^ and *Brettanomyces* 0–400 CFU/m^3^ [61,75].

In healthy buildings, the majority of micro-organisms present indoors originate from the outdoors. Therefore, the species found and their relative proportion frequently mimic the outdoor microbiota at a given moment. In temperate climates, fungi are found in higher abundances outdoors than indoors and vary along seasons. Indoors, *Cladosporium* spp. dominates the mycobiota, followed by *Penicillium* spp., non-sporulating fungi (*mycelia sterilia*) and *Aspergillus* spp. [80,88,89,90].

In general, the indoor air fungal contamination in wineries was higher than that of outdoor samples, and fungal count fluctuations depended upon the type of monitored environment [91]. In the 36 wineries and at all sampling sites (71), the predominant genera in indoor air were *Penicillium* (45.1%), *Aspergillus* (20.5%) and *Cladosporium* (11.5%). *Botrytis cinerea* spores occurred more frequently (*p* < 0.01) in production areas [67], a pattern also detected by Simeray and collaborators, who reported an elevated number of *B. cinerea* from grape pressing in the making of “vin de paille” [80].

While studying the mycobiota in the air at six wineries in Hungary, the indoor samples were dominated by penicillia, with a total of 12 species (including the genera *Rasamsonia* and *Talaromyces*) present. The most frequent species was *Penicillium spinulosum*, detected in five of the six cellars at concentrations above 13,000 CFU/m^3^. Almost half of the *Penicillium* species (47%) were found in just one cellar, among them, *Penicillium adametzioides*, *Penicillium solitum* and *Penicillium thomi* [73]. The indoor microbiota was more diverse than outdoors, and indoor samples depended on sample proximity [73,91]. Also, the species diversity in the air was correlated with the age of the cellars. Among the environmental conditions, elevation, age, reconstruction time of cellars, indoor ethanol concentration and the number of chimneys influenced the mycobiota of the examined wine cellars to a certain degree [73].

In 12 wine cellars, the dominance of the three fungi genera, *Penicillium*, *Cladosporium* and *Aspergillus*, seemed winery-dependent. Simeray and collaborators [80] reported (a) wineries where *Penicillium* dominated, the most frequent case (*P. glabrum*, *P. implicatum*, *P. dierckxi*, *P. waksmanii*, *P. brevicompactum*, *P. roqueforti*, followed by *P. expansum*); (b) wineries where *Cladosporium* dominated, a less common case, especially associated with old facilities (*C. sphaerospermum*, followed by *C. herbarum* and *C. cladosporioides*); and (c) wineries where *Aspergillus* dominated (*A. versicolor* and *A. restrictus*), also rare. Occasionally, other taxa were significant, such as *Botrytis cinerea*, *Phoma* spp. and *Oidiodendron griseum*. Interestingly, thermotolerant species (growing at 37 °C) were exclusively found in old cellars (*Aspergillus fumigatus*, *A. niger*, *Eurotium amstelodami*, *E. intermedium*, *Penicillium decumbens*, *P. pinophilum*, *Trichoderma koningii*, *P. variabile* and *Scopulariopsis brevicaulis*).

Yeasts were also detected in winery indoor air. In general, yeast concentrations in indoor air are low and vary among seasons (higher during harvest), throughout winemaking (higher in alcoholic fermentation) and wine handling [84,85]. In a survey of yeasts found in the air of different areas in three wineries over one year, *Cryptococcus* and *Aureobasidium* were the genera permanently present in the air of all the wineries during the sampling period [86]. Some yeast genera, such as *Sporobolomyces* and *Williopsis*, were present in two of the three wineries. Also, different yeasts appeared in distinctive winery areas. For instance, *Cryptococcus*, *Sporidiobolus* and *Rhodotorula* were permanently present in the air in the bottling line of the three wineries during the survey. On the contrary, *Candida*, *Saccharomyces*, *Kloeckera*, *Torulaspora* and *Sporobolomyces* were always absent from the air in the bottling line [85]. *Brettanomyces*/*Dekkera* yeasts were isolated from the wineries’ air (mean values 2 MPN/m^3^ air) mainly from air in the casks in two of the three wineries (5 and 18 MPN/m^3^ air), when wine containing this yeast was being handled [84]. This yeast, associated with wine defects, was also detected by Connell et al. (2002) [81] in the air of four areas (crush, tank, cask and bottling rooms) in one winery at 12 CFU/m^3^ and 400 CFU/m^3^ air in the bottling and in the pressing areas, respectively. Most of the isolated yeasts were members of the non-*Saccharomyces* group.

*Saccharomyces cerevisiae* was only detected in the air in the vinification area during alcoholic fermentation [85]. Another study of yeast levels in the air of four areas of a winery (vinification, bottling line, cask and bottle cellar) conducted over one year on a monthly basis confirmed that the levels of yeasts present in the air were low, except at harvest time and in the vinification area, where the population of *S. cerevisiae* in the air surrounding the fermentation vats achieved 180 MPN/m^3^ air. Except during the vinification stage, in the rest of the samples, all yeasts were non-*Saccharomyces*. The highest yeast levels and diversity during the year were reached in the bottling area, which makes this area the most vulnerable point for air contamination [85]. Despite the low yeast values reported in wineries indoors, in the air of Hungarian wineries, the species *Candida membranifaciens* and *Debaryomyces subglobosus* appeared in two cellars in high numbers: 13,000 CFU/m^3^ and 760 CFU/m^3^, respectively [73].

Most of the studies of indoor air bacteria point out that the bacterial load is identical between outdoors and indoors [92] and that the bacterial indoor diversity is dominated by Gram-positive *Actinomycetota* (formerly *Actinobacteria*) and *Firmicutes* over Gram-negative *Proteobacteria* [93,94]. In addition to fungi, the microbial air community in wineries includes LAB, within *Firmicutes*, and their presence in the air of wineries is influenced by the activity taking place and the moment of fermentation [61,75].

An investigation into the bacterial diversity and concentrations in the indoor and outdoor environments of three types of industrial installations, including a winery, in Malaysia showed that *Micrococcus luteus* (~300–470 UFC/m^3^ air) and *Kytococcus sedentarius* (~140 UFC/m^3^ air) were the predominant bacterial species identified in indoor air (Table 3 and Table 4). Both of these Gram-positive cocci are opportunistic pathogens.

It is also worth mentioning that the percentage of cultivable micro-organisms in the air is quite low. Zollinger and colleagues, when quantifying the particles suspended in the air using laser particle sizers and comparing them using the impaction sampler in known selective media for bacteria and fungi, found that culturable micro-organisms only accounted for 0.1–0.2% in relation to the total number of airborne particles emitted [79]. The survival of air indoor micro-organisms and their subsequent growth are closely related to environmental conditions, such as temperature, humidity and the culture media [75,95].

To date, the definition of a limit for air indoor micro-organisms corresponding to a specific level of unsafe microbial contamination remains unresolved [76]. In wineries, and despite the few studies focused on the composition of indoor air microbiota, there is a tendency to prevent the growth of micro-organisms in these environments [67]. In the context of healthy and sustainable buildings, the European Community Board [96] prepared a report on the indoor air quality of private houses, non-industrial workplaces and public buildings, where the impact on man was categorized as “very low” (<50 CFU/m^3^), “low” (50–100 CFU/m^3^), “medium” (100–500 CFU/m^3^) and “high” (>500 CFU/m^3^). In wineries, the number of fungi and bacteria per m^3^ air can reach 146,000 UFC/m^3^ and 485,000 UFC/m^3^, respectively (cf. Table 4), values much higher than those mentioned above. In other food industries, such as the meat industry, bakeries and the dairy industry, the proposed limits are lower depending on the products, the processing room, the hygiene and the target micro-organism (e.g., [76,97,98]). However, all the proposed limits lack consistency, highlighting the need for scientifically based and context-specific limits [99].

## 4. Microbial Risks in Wineries

Many of the numerous microbial species found in wineries (Supplementary Table S1) affect the quality of the wine or the winery workers’ and visitors’ health. Also, the winery’s environment—dark and damp—makes these places particularly vulnerable, fostering microbial growth on winery surfaces and in the air.

### 4.1. Risks to the Wine

The natural wine microflora includes many species—bacteria, yeasts and molds—that can spoil the wine, translated into undesirable aromas and flavors. Two bacterial groups are frequently involved in wine spoilage: lactic acid (LAB) and acetic acid (AAB) bacteria. The most frequent metabolites produced by AAB are acetic acid, ethyl acetate and acetaldehyde, which, respectively, confer vinegar, nail polish and bruised apple aromas (Table 5). Also, within the bacterial community, LAB are responsible for volatile substances associated with unpleasant odors (e.g., caged mouse) or tastes (e.g., crushed geranium leaves or acetic acid). Some species of *Lactobacillus* and *Oenococcus* also produce diacetyl, which gives a buttery, nutty, caramel taste, and ethyl carbamate, an organic molecule that has a saline/bitter taste. Both acetaldehyde and ethyl carbamate, also known as urethane, are probable human carcinogen organic molecules [100]. Usually found in wineries, *Bacillus* species (*B. subtilis*, *B. circulans*, *B. coagulans*) and *Pediococcus* (usually *P. parvulus*) can produce long-chain polysaccharides or β-D-glucan, increasing wine viscosity and giving an oily appearance [101].

Yeasts such as *Candida*, *Kloeckera*/*Hanseniaspora*, *Pichia* (*P. membranifaciens*, *P. anomala*) and *Saccharomycodes* produce acetic acid and ethyl acetate, which give vinegar and nail polish aromas. Also, many yeasts from the *Saccharomyces*, *Saccharomycodes*, *Schizosaccharomyces*, *Zygosaccharomyces* genera are responsible for film or pellicle formation, cloudiness and sediment (Table 5).

Some of the mold species found in wineries can produce volatile organic compounds (VOCs) responsible for bad tastes/aromas in wines. *Penicillium expansum*, *B. cinerea* and some other fungal species are reported to produce trans-1,10-dimethyl-trans-9-decalol, also known as geosmin [102].

**Table 5:** Winery micro-organisms that cause defects in wine. (Adapted from [103,104,105,106,107]). * toxic [108]; ** reasonably anticipated to be human carcinogen [100].

Taxa (Genus/Species) or Group)	Metabolites	Instability/Flavors/Risks/Faults
**Bacteria**		
*Bacillus* (*B. subtilis,* *B. circulans*, *B. coagulans*)	Long-chain polysaccharides	Sediment/haze formation, ropiness, viscous
LAB	Acetic acid	Vinegar
*Lactobacillus*	Acrolein *	Bitterness
*Lactobacillus*, *Oenococcus*	diacetyl (2,3-butanedione)	Buttery, nutty, caramel
*Lactobacillus*, *Pediococcus*	2-Ethoxy-3,5-hexadiene	Crushed geranium leaves
*Lactobacillus*, *O. oeni*	2-Acetyl-tetrahydropyridine, 2-Ethyl-tetrahydropyridine	Caged mouse/mousy
*Oenococcus*	Mannitol	Viscous, sweet
*Leuconostoc*, *Pediococcus*	β-D-glucan	Viscous and thick
AAB, LAB	Acetic acid, Ethyl acetate	Vinegar, solvent-like aroma, nail polish removal
AAB (*Acetobacter*, *Gluconobacter*)	Acetaldehyde **	Bruised apple, sherry-like, nutty
*O. oeni*	Ethyl carbamate **	Saline, bitter taste
*Streptomyces*	Guaiacol, 4-methylguaiacol	Smoked, phenolic and medicinal odors
**Yeasts**		
*Candida*	Acetic acid, ethyl acetate	Vinegar, nail polish removal,
*C. boidinii* *P. guilliermondii*	4-vinylphenol 4-vinylphenol, 4-ethylphenol	Barnyard, medicinal, band-aids, mousy
*Brettanomyces*/*Dekkera*	4-ethylphenol, 4-ethyl guaiacol, 2-acetyl-tetrahydropyridine, 2-ethyl-tetrahydropyridine	Barnyard-like, horsy, wet wool, sweaty saddle, medicinal, mousy
*Hansenula*	Acetic acid, ethyl acetate	Vinegar, nail polish removal, film formation
*Kloeckera*/*Hanseniaspora*	Acetic acid, ethyl acetate	Vinegar, nail polish removal, film formation, yeasty and estery aromas
*Pichia* (*P*. *membranifaciens*, *P*. *anomala*)	Ethyl acetate	Film formation, nail polish removal
*Saccharomyces*	NA	Cloudiness, sediment, yeasty aroma/flavor
*S. cerevisiae*	Hydrogen sulphide	Rotten-egg off-flavor
*Saccharomycodes*	Acetoin, ethyl acetate, acetic acid	Film/sediment formation, gas production, vinegar, nail polish removal
*Schizosaccharomyces*	NA	Sediment
*Zygosaccharomyces*	NA	Haze or deposit after bottling
**Molds**		
*B. cinerea*	1-octen-3-ol, 1-octen-3-one	Metallic, fresh mushrooms
*B. cinerea*, *P. expansum*	Geosmin (trans-1,10-dimethyl-trans-9-decalol)	Dump hearth, humus, dump cellar
*Aspergillus* (*A. niger*), *Paecilomyces*, *P. chrysogenum*, *P. glabrum*, *Mucor racemosus*, *Trichoderma viride*	2,4,6-trichloroanisole (TCA) 2,3,4,6-tetrachloroanisole (TeCA) 2,4,6-tribromoanisole (TBA)	Cork taint, wet dog Moldy cellar odor Moldy and mushrooms

NA—Not applied; LAB—Lactic acid bacteria (e.g., *Lactobacillus*, *Leuconostoc*, *Oenococcus*, *Pediococcus*); AAB—Acetic acid bacteria (*Acetobacter*, *Gluconobacter*).

*Penicillium* and *Trichoderma* species may contribute to the formation of 2,4,6-trichloroanisole (TCA) [108]. Geosmin is an earthy musty, and TCA is recognized by cork taint, the most frequent wine fault (“wet dog” smell), making wine unpalatable. TCA has been detected in the air of Austrian wine cellars with visible moldy patches [67].

### 4.2. Risks to the Wine Consumers, Workers and Visitors

Biogenic amines (BAs) are low-weight organic bases with biologic activity. They have important functions in human physiology, such as regulation of the immunological system, gastric acid, blood pressure, neurotransmission and cell cycle [109,110,111,112,113]. However, exposure to high concentrations of BAs can induce adverse reactions, such as nausea, allergic reactions, headaches, rashes, hallucinations and changes in blood pressure [111,112].

In wine, most of the BAs are produced by LAB and some yeast species (Table 6), but this ability has also been reported for other species isolated from wines, such as *Enterococcus faecium* and *Staphylococcus epidermis* [114,115]. Most of the BAs result from amino acid decarboxylases, and, in wines, histidine, tyramine, cadaverine and putrescine are the most frequently reported, with the last two found at the highest concentrations [116]. Among BAs, histamine is the most important compound related to food-borne intoxications due to its strong biological activity. It is well known that allergy-like food poisoning is mediated by histamine, causing inflammatory and anaphylactic reactions, such as headaches, rashes, flushing, itching, swelling, runny or blocked nose, irregular heartbeat, diarrhea, nausea, vomiting or abdominal pain [117].

Many fungal species isolated from the winery environment can produce mycotoxins, secondary metabolites that, in animals (humans included), are responsible for acute and chronic symptoms: allergies, liver cancer, nephropathy, genotoxicity and teratoxicity. Among these molecules, aflatoxins and ochratoxin A (OTA) are produced by many *Aspergillus* and *Penicillium* species; fumonisins are produced by *Fusarium* species and some *Aspergillus* species; and *Alternaria* mycotoxins, mainly alternariol, are produced by *Alternaria alternata* and *B. cinerea* (Table 6). Aflatoxins and OTA are known to be human carcinogens [100].

In wine, the most frequent mycotoxin is OTA, which inhibits the phenylalanyl-tRNA synthetases by competing with phenylalanine and decreases the level of phosphoenolpyruvate carboxykinase, interfering with gluconeogenesis [120]. Due to its high frequency, OTA is the only mycotoxin with an established maximum level in wine (2 μg/L) [121]. In a study with 19 wines from Spain, France and Poland, all of the wines exceeded the mycotoxin limits. Also, the levels of OTA contamination are country- and farming-method-dependent [122]. Contrarily, in 100 samples from wines purchased on the Portuguese market, 5% of the samples were contaminated with OTA at concentrations within the limits of detection (LOD) and quantification (LOQ) [123]. Similar results were reported for Chile, where OTA was detected only in 2.9% of all samples (n = 400) [124]. Red wines have higher OTA contents [122,123,125]. Fumonisins, produced by *Fusarium* spp., *A. niger* and *A. welwitschiae* [126,127], species frequently found in wineries (see Section 3), interfere with sphingolipid biosynthesis by inhibiting ceramide synthase [128]. Maximum tolerable levels for fumonisins are established for maize products only at levels > 800 µg/L, depending on the product (except for baby food, which is 200 µg/L). Several studies reported the contamination of wines with fumonisin B2 (FB2), with levels up to 25 µg/L [129,130]. Although exhibiting low levels, these studies indicate a potential risk of FB2 exposure to the wine consumer.

Along with wine contamination by mycotoxigenic species, the inhalation of fungal spores or propagules can expose animals to toxins. The risk of indoor mycotoxigenic contamination depends on the RH, water leaks that can lead to mold outbreaks and the quality of ventilation [131]. For example, *Aspergillus ochraceus* was isolated from wheat in a silo after a worker and his wife experienced acute kidney failure when cleaning a granary. They were probably exposed to ochratoxin A [132].

Although direct risk of microbial contamination in wine is considered low, some researchers have reported the presence of pathogenic species. In Spain, two strains of *Staphylococcus epidermidis*, a common member of the human skin microbiota, were isolated from a histamine-rich Tempranillo red wine from Ribera de Duero. One of the strains was capable of producing histamine, cadaverine and putrescine, both in synthetic medium and in grape must [114]. *Enterococcus faecium* was isolated from red wines in Italy [115], Spain (sampled in 2011 vintage year in a winery located in Castilla-La Mancha) [64] and in Turkey [133]. Also, *E. faecium* was isolated from must undergoing MLF in Spain, and its survival in wine was ethanol-dependent, with some strains able to survive at ethanol 10% (*v*/*v*) for more than 30 days [64]. This Gram-positive species also lives in the vertebrate gut and is often associated with or responsible for nosocomial infections, particularly among the elderly or people with chronic diseases, and it exhibited antibiotic resistance [134]. Also, *E. faecium* produces tyramine, a BA involved in toxic reactions [115].

Nisiotou and collaborators reported high bacterial diversity from spontaneous fermentation of botrytized grapes in Greece. Among 253 bacterial isolates, 8 of the 11 species identified were considered pathogenic or opportunistic pathogens, such as *Citrobacter freundii*, *Enterobacter ludwigii*, *Klebsiella oxytoca*, *Pantoea dispersa*, *Providencia rettgeri*, *Serratia marcescens*, *Staphylococcus epidermidis* and *Tatumella ptyseos* [135]. Both *E. ludwigii* and *S. epidermidis* can cause human infections, with strains resistant to antibiotics [136,137].

In an investigation into the bacterial diversity and concentrations in the indoor and outdoor environments of industrial installations, including a winery, it was shown that *Kytococcus sedentarius* and *M. luteus* were the predominant indoor air bacteria (above 800 UFC/m^3^ air). *Micrococcus luteus* causes pneumonia in immunocompromised individuals, and species of *Kytococcus* are emergent and opportunistic pathogens among immunocompromised individuals, children and the elderly [86].

Despite the high microbial diversity and the presence of pathogenic or opportunistic pathogenic species in wineries, there is a deep-rooted belief that their impact on the health of winery users is low or even non-existent. But the presence of viable cells of *E. faecium*, *E. ludwigii*, *S. marcescens*, *S. epidermis* in musts and wine [114,115,135] and other pathogenic or opportunistic species during or after fermentation (*K. oxytoca*, *P. dispersa*, *P. rettgeri* and *T. ptyseos*) highlights the ability of these bacteria to cope with harsh environmental conditions and their potential hazard to human health.

The high fungal counts observed in the bottling areas of industrial producers (Switzerland) and in the fermentation areas of family-run wineries (Italy) of *A. niger*, *A. ochraceus*, *A. terreus*, *C. cladosporioides*, *A. alternata*, *Penicillium chrysogenum*, *Penicillium citreonigrum*, *Penicillium crustosum* and *Penicillium viridicatum* can pose a risk to winery workers or visitors [91]. Fungal allergens can be found in fungal spores, hyphae and yeast forms, and indoor fungi, mainly those of the Ascomycota and Basidiomycota phyla, are significant sources of allergens. In Ascomycota, species of *Alternaria*, *Aspergillus*, *Cladosporium*, *Botrytis*, *Epicoccum*, *Fusarium* and *Penicillium* are of clinical importance, as they release large quantities of spores. The yeasts *Candida*, *Saccharomyces* and *Rhodotorula*, as well as the molds *Mucor* and *Rhizopus*, can also produce allergens [138]. All of these fungi are present in winery environments (Supplementary Table S1) and can provoke asthma, rhinitis, hypersensitivity pneumonitis and allergic bronchopulmonary mycoses. In addition to allergens, some molds (e.g., *Penicillium*, *Aspergillus*, *Alternaria*, *Fusarium*, *Stachybotrys*) can produce mycotoxins. These toxins can affect humans and animals after ingestion of contaminated food, as previously mentioned, but also via inhalation and dermal contact [139,140].

Probably the most important hazard that workers face in wineries is carbon dioxide [141,142], a colorless and odorless gas at low concentrations produced during fermentation. In confined spaces, winery workers can easily be exposed to CO_2_ above 0.5% (5000 ppm), which can be toxic to humans, provoking a sudden shock in the nerve centers and potentially resulting in syncope and death. Employer awareness of the risk, adequate ventilation, CO_2_ detectors associated with visual and sound alarms, adequate instructions to employees of the risk and its prevention and adequate signage in the riskiest places will reduce the probability of accidents caused by this gas [143].

## 5. Challenges in Wineries

### 5.1. Microbiology of Wineries and Climate Change

In vines under severe stress (heat and drought), a decrease in photosynthetic productivity is observed, which can significantly influence the quality of the grapes and, by extension, the quality of the wine. High temperatures influence both the primary and secondary metabolisms in vines. In grapes, sugar content and the proportion of hexoses, the anthocyanins’ composition and concentration, the amino acids’ levels and profile and the synthesis of phenolics and volatile compounds are affected by heat stress [144,145,146].

Heat, water activity and the combination of both can affect yeast communities in grapes, namely the proportion of *Saccharomyces*/non-*Saccharomyces*, which is consequential for wine typicity and its aromatic profile [147]. In Table 7, it is evident that yeast growth is highly hindered above 34 °C.

Obviously, the impact of climate and weather on the vineyard and grape chemistry will also be reflected in the winemaking. Despite grape berries accumulating less sugars and having high glucose/fructose ratio after exposure of grapevines to heat stress (40/20 °C day/night temperatures) [144], under drought conditions, sugars may increase in grapes. The accumulation of sugars in grapes may be due not to increased photosynthesis but to dehydration of the berries [148]. Musts with high sugar concentration are stressful to yeasts, interfering with their growth or even provoking lysis, stuck alcoholic fermentation and promote the appearance of fermentative by-products, such as glycerol and acetic acid [149]. Also, high ethanolic levels can affect the growth of yeasts. Above 14% of ethanol, only 5 (38.5%) of the 13 strains of *S. cerevisiae* grew, and at 16% of ethanol, only one strain was able to grow. At 15% of ethanol, strain growth decreased [150]. Malolactic fermentation is also affected by high levels of ethanol in musts, since it compromises LAB cell membrane integrity [151]. These setbacks can delay wine stabilization or make winemaking more expensive.

**Table 7:** Temperature growth range for some important yeast species associated with winemaking [152]. Legend: (+) growth; (−) no growth; (v) variable; (nd) no data.

			Growth at		
Yeast Species	19 °C	25 °C	34 °C	37 °C	40 °C
*Candida apicola*	+	+	v	−	−
*Candida bombi*	+	+	+	+	−
*Candida parapsilosis*	+	+	+	+	v
*Candida stellatta*	+	+	v	−	−
*Candida sake*	+	+	−	−	−
*Candida vini*	+	+	−	−	−
*Debaryomyces carsonii* *	+	+	nd	v	nd
*Debaryomyces hansenii* **	+	+	+	+	nd
*Dekkera bruxelensis*	+	+	+	+	v
*Hanseniaspora guilliermondii*	+	+	+	+	−
*Hanseniaspora uvarum*	+	+	nd	−	−
*Issatchenkia occidentalis*	nd	+	+	+	−
*Kloeckera lindneri*	+	+	−	−	−
*Kluveryomyces thermotolerans*	+	+	+	v	nd
*Metschnikowia pulcherrima*	+	+	nd	v	nd
*Pichia anomala*	+	+	nd	v	nd
*Pichia membranifaciens*	+	+	nd	v	nd
*Saccharomyces bayanus*	+	+	v	−	−
*Saccharomyces cerevisiae*	+	+	v	v	v
*Schizosaccharomyces pombe*	+	+	nd	−	nd
*Torulaspora delbrueckii*	+	+	nd	v	nd
*Zygosaccharomyces bailii*	+	+	nd	v	nd

* Synonyms: *Pichia carsonii* or *Priceomyces carsonii*. ** At a temperature of 36 °C, some strains lose their full viability within 12 h [19].

In addition, heat and water can favor pests, interfering with the vine and grape microbiota, which may influence winemaking and the chemical composition of the wine, potentially leading to the accumulation of mycotoxins and BAs in wine [153,154,155]. These can affect both the quality and the safety of the wine. For instance, heat and drought/low water activity in the vineyard can favor thermotolerant and xerophilic fungi, such as *A. fumigatus*, *A. niger* and *Aspergillus flavus*, all of them mycotoxigenic and allergenic species [75,82]. Nevertheless, the production of aflatoxins can be affected by the matrix, as in the case of *A. flavus* isolates isolated from grapes in Spain that were unable to produce both aflatoxins B1 and B2 in grape-based media under different temperature (28–37 °C) and water activity (0.99–0.90) combinations [150]. The increased level of mold contamination of grapes may further increase the need for sulfites, which in turn can affect the wine microbiota. The biotypes of *S. cerevisiae* are greatly affected by sulfites, even at low concentrations (40 mg/L) [150].

Changes in grape chemistry and microbiology bring technological, architectural and energetic challenges (see point 5.2). As the grapes are harvested earlier, when temperatures are higher (August/September), they arrive warmer at the winery, whose interior is hotter, making fermentation challenging. The temperature of fermentation has an impact on wine composition. Wines fermented at 16 °C, 20 °C and 27 °C significantly differed in terms of BAs, citric acid, acetic acid and total sulfites. Sulfites decreased at higher temperatures, contrary to BAs, which increased, while volatile compounds’ concentrations varied depending on the molecule [156]. The temperature of fermentation also influences other wine characteristics. As the temperature increases, the concentration of fusel alcohols, polysaccharides and glycerol increases, while that of esters decreases [157].

The kinetic and thermal models developed for alcoholic fermentation in winemaking conditions, with the climate-change-related variables “must sugar concentration” and “exterior temperature”, suggest a linear increase of approximately 5% in total cooling energy requirements for every increase of 1 °C in exterior temperature or 10 g sugar per must liter [158]. Therefore, to maintain fermentation at low temperatures to preserve the aromas, energy costs will be inflated. This is where a good installation design can contribute to the environmental and financial sustainability of the winery.

### 5.2. Wineries’ Design

Despite the small number of bibliographical references on the design of wineries, the study performed by Goméz et al. [99] in the winemaking region of La Rioja, Spain, enables the identification of four different classes of wineries. (i) Class A—wineries such as cooperatives that generally make wine to sell in bulk to other wineries with a small aging area; (ii) Class B—industrial scale wineries with a rational layout and areas for wine production, aging and bottling. This class can be divided into two subclasses: those with an area under 2500 m^2^ (Class B-1) and those with an area over 2500 m^2^ (Class B-2); (iii) Class C—unconventional industrial wineries, where the design is not only based on the functionality required for undertaking the winemaking processes but also on criteria such as leisure activities and esthetics. This type of winery is usually large (over 2500 m^2^).

Regarding the authored wineries, a study performed by San-Antonio-Gómez and collaborators evaluating 21 wineries located worldwide showed that, in Europe, there is a predominance of wineries with two floors and with a greater variation in aging areas, many of them underground, as opposed to the rest of the world, where none was reported. Also, in Europe, the use of natural and mixed hygrothermal conditioning is higher than in the rest of the world. Concerning materials, for the wineries located in Europe, concrete is the elected material for façade covering, whereas stone and metal panels are more common in other countries outside of Europe [159]. The evaluated wineries were built for producing excellent wines, providing an image of modernity through their architecture and improving their environmental and energetic sustainability. In the context of climate change, it is vital to improve energy efficiency (like insulating green roofs), water conservation, rainwater harvesting and reuse, a high-efficiency heating, ventilation and air conditioning (HVAC) system and a design to allow the use of gravity in winemaking [159]. The use of environmentally friendly materials maximizing efficiency and minimizing waste and carbon footprint reduction during and after construction are now common practices [160]. For example, fermentation can take place in tanks made of many materials. Stainless-steel tanks are currently the most widespread, as they maintain the freshness of the grapes, do not transfer flavors to the wine and provide perfect hygiene. When using cement tanks, their maintenance is essential. On the other hand, fermentation in wooden barrels gives unmistakable flavors and aromas in addition to “softening” the tannins of the resulting wine. One of the disadvantages of using wood is that it is very difficult to clean, forming a favorable habitat for the development of certain contaminating micro-organisms. For molds, winery design seems to be the fundamental factor for microbial air dissemination. In areas with low ventilation and air renewal and with high RH, the mold presence is high [83]. These authors found that in a survey of air microbiology in a wine cellar in Logroño, La Rioja (Spain), some environmental parameters differed between the two cellar levels (ground and underground). Also, the studies performed by Bokulich et al. [65] in the USA and by Varela et al. [66] in Australia are interesting examples of the microbiota and microbiome variations in the different areas of wineries over time (see Section 2).

## 6. Conclusions and Future Perspectives

Microbial diversity differs between wineries, and in each winery, in its various niches, but there appears to be a resident community of micro-organisms common to all wineries (see Table S1). The grapes, grape musts, surfaces in contact and air are interconnected microbial niches [11,47]. Winery surfaces and equipment favor microbial growth, with a resident community that, in each winery, appears to depend on the type of surfaces, the activity taking place and the season [11,19,46,47,66,68]. The winery microbial community may have an impact on the quality of the wine, on the health of its consumers or on the health of winery workers and visitors [64,86,91,103,104,105,106,114,115,118,119,135].

Classic microbial methods, albeit important for studying a specific micro-organism and its metabolism, are limited and time-consuming [161]. Knowledge of the fungal diversity during winemaking increased with the greater use of NGS methods [30,46,53,57,65,66,162], an approach that detects all species present in complex environments. Third-generation metagenomic sequencing opens up new possibilities for microbial monitoring. Environmental DNA sequencing (eDNA) using high-throughput sequencing and metabarcoding methods is revolutionizing the monitoring of biological diversity [163]. For instance, the latest MinION Nanopore sequencing can be used in the field [164].

Flow cytometry, also a culture-independent method, can be used to quantify microbial cell densities, offering the opportunity to gain insights into microbial viability, physiology and metabolism [161]. The monitoring and identification of specific species can be visualized using fluorescence probes and the fluorescence in situ hybridization (FISH) technique, as performed in micro-vinification studies [165]. This technique has recently been adapted for identifying the wine spoilage yeast *Dekkera bruxellensis* using DNA sequences targeting RNA probes in the wine environment [166]. Also, primers targeting specific genes using quantitative PCR (qPCR) are being used for detecting spoilage organisms, ensuring the stability and quality of the wine [167]. Both FISH and qPCR techniques are highly sensitive and have as their main disadvantage the need for expensive equipment [161].

Increasing the knowledge of microbial communities from grape to wine will be essential to mitigate the effects of climate change in wine production. The combination of heat and water activity affects grape quality and its microbiome, as well the winery microbiome, having consequences for the characteristics of the wine. Additionally, heat and water activity drive pests and favor thermotolerant and xerophilic fungi (e.g., *A. fumigatus*, *A. niger*). Some of these species are capable of producing mycotoxins and BAs that accumulate in wine [153,154,155]. Non-*Saccharomyces* species have been studied in order to overcome some of these constrains [24,147,168].

In wineries, the control of temperature and RH is essential, both during fermentation and wine storage. With the prospect of extreme climatic events, such as heat waves and droughts/floods, maintaining the temperature and RH within wineries is essential for microbial stability and to preserve the quality and safety of the wine.

## Figures and Tables

**Table 3:** Micro-organisms in winery air: winery type, concentration (MPN/m^3^ air or CFU/m^3^ air) mean and range per culture medium from works cited (2001–2022), ordered by publication date.

Winery Type	RT (°C)	RH (%)	Micro-Organism	Concentration Mean (CFU or MNP per m^3^)	Concentration Range (CFU or MNP per m^3^)	Country [Reference]
Old, modern and “Vin jeune” cellars	~9.0–16.0	40–60	Mesophilic molds Xerophilic molds	MEA: 665 MESA: 826	MEA: 57–2422 MESA: 98–2547	France [80]
Medium-sized winery	NR	NR	*Brettanomyces*	NR	BSE: 12–400	USA [81]
Indoor processing facility	NR	NR	Bacteria Bacteria (Gram−) Fungi	NR NR NR	TSA: ~500–485,000 MCA: 0–180 MEA: ~200–146,000	[79]
Old subterranean House-family Modern air-conditioned	~9.0–13.0 NR ~9.5–15.3	~85–93 NR ~72–81	Molds	MEA: 4769 MEA: 4293 MEA: 357	MEA: 848–12,050 NR NR	Austria [82]
Winery (only red wines)	NR	NR	Molds Yeasts LAB		CZA: 500–10,000 CGA: 0–1000 MRS: 0–500	Spain [75]
Winery in La Rioja	NR	NR	LAB	MRS: 18	MRS: 0–328	Spain [61]
Brick and concrete wineries	13.0–18.0	39–85	Mesophilic molds Xerophilic molds	MEA: 1300 DG18: 1400	MEA: 35–12,000 DG18: 110–14,000	Austria [67]
Modern winery (four areas) in La Rioja	10.3–23.5	45–86	Molds	CZA: 2053	CZA: 355–29,000	Spain [83]
Three modern wineries (four areas) in La Rioja	NR	NR	Yeasts total *Brettanomyces*	CGA: NP DBDM: 1	CGA: 0–180 DBDM: 0–18	Spain [85]
Modern winery (four areas, 12 months) in La Rioja	NR	NR	Yeasts	CGA: 20 DBDM: 0	CGA: 0–240 DBDM: 0	Spain [84]
Winery (three areas, 2 years) in Castilla-La Mancha	NR	NR	Yeasts LAB	NR NR	CGA: 87–2300 MRS: <100–1800	Spain [64]
Old (subterranean) and modern wine cellars	13.7–17.7	52.4–89.5	Molds	RBAC: 2788 MEA: NP	RBAC: 75–8190 MEA: NR	Hungary [73]
Underground cellar, covered by earth	3.9–15.1	82–93	Molds	NR	NR	Romania [87]

RT—Room temperature; RH—Relative humidity. MEA—Malt extract agar; MESA—MEA supplemented with 20% sucrose; NR—Not reported; BSE—*Brettanomyces* selective agar; TSA—Tryptone soy agar; MCA—MacConkey agar; LAB—Lactic acid bacteria; CZA—Czapek agar; CGA—Chloramphenicol glucose agar; MRS—De Man–Rogosa–Sharpe agar; DG18—Dichloran glycerol agar; DBDM—Selective medium for *Brettanomyces*; RBAC—Askew agar with Rose Bengal agar and chloramphenicol.

**Table 4:** Microbial diversity and abundance (in brackets, when reported) in winery air, enhancing the dominant group or taxa.

Winery Type	Micro-Organism Type	Dominant Group/Genera/Species (%)	Other Genera/Species (≤10%)	Reference	Country
Twelve wine cellars (Arbois region)	Molds	*Penicillium*, *Cladosporium*, *Aspergillus*	*Botrytis cinerea*, *Zasmidium cellare*, *Emericella nidulans*, *Geotrichum*, *Mucor racemosus*, *Mycosphearella*, *Oidiodendron cereale*, *O. griseum*, *Spiniger meineckellus*, *Wardomyces inflatus*	[80]	France
Old subterranean House-family Modern air-conditioned	Molds	*Penicillium* (77%), *Aspergillus* (15%) *Penicillium* (78%), *Aspergillus* (16%) *Penicillium* (50%), *Aspergillus* (20%)	*Exophiala*, *Trichoderma* - *Clasdosporium*, *Phialophora*, *Phoma*, *Trichoderma*, *Ulocladium*	[82]	Austria
Winery (pre-, during and post-harvest)	Molds Yeasts	*Penicillium* (20–80%), *Aspergillus* (10–50%), *Botrytis* (20–40%) Non-*Saccharomyces* (50–100%), *Saccharomyces* (20–100%)	- -	[75]	Spain
Winery (pre-, during and post-harvest)	LAB	*Oenococcus oeni* (15–100%)	*Leuconostoc mesenteroides*, *Pediococcus pentosaceus*	[61]	Spain
Thirty-six (2×) wine cellars (from 20 Styrian vintners)	Molds	*Penicillium* (45.1%), *Aspergillus* (20.5%), *Cladosporium* (11.5%)	*Acremonium*, *Alternaria*, *Aureobasidium*, *Botrytis*, *Chrysonilia*, *Emericella*, *Epicoccum*, *Eurotium*, *Fusarium*, *Geotrichum*, *Moniliella*, *Mucor*, *Phanerochaete*, *Phoma*, *Rhizopus*, *Scopulariopsis*, *Schizophyllum*, *Stachybotrys*, *Trametes*, *Trichoderma*, *Trichothecium*, *Ulocladium*, *Wallemia*	[67]	Austria
One winery (from February to December, in four places)	Molds	*Penicillium* (up to 90%), *Aspergillus* (up to 50%)	*Alternaria*, *Botrytis*, *Clasdosporium*, *Fusarium*, *Paecillomyces*, *Trichoderma*	[83]	Spain
Three wineries, 10–40 years old (over four seasons, in four places)	Yeasts	*Cryptococus* (23–73%), *Aureobasidium* (6–25%), *Saccharomyces* (6–10%), *Sporidiobolus* (1–16%)	*Debaryomyces*, *Candida*, *Kloeckera*, *Pichia*, *Rhodotorula*, *Torulaspora*, *Sporobolomyces*, *Sporidiobolus*, *Bretanomyces*, *Arthroascus*	[85]	Spain
One winery (from February 2008 to January 2009) in four areas (cask and bottle aging cellars, vinification and bottling)	Yeasts	*Cryptococcus* (30.3%), *Sporidiobolus* (20.1%), *Aureobasidium* (12.1%)	*Candida*, *Debaryomyces*, *Williopsis*, *Rhodotorula*, *Pichia*, *Sporobolomyces*, *Saccharomyces*, *Bullera*, *Torulaspora*	[84]	Spain
One winery (over 2 years, in three places, one season per year)	LAB	*L. mesenteroides* (31–56%), *Pediococcus acidilactici* (0–29%), *O. oeni* (0–24%), *Lactobacillus casei*/*paracasei* (1–24%), *Lactobacillus plantarum* (0–11%)	*Enterococcus* sp., *Lentilactobacillus hilgardii* *Lactococcus lactis* spp. *hordniae, Lactobacillus brevis*, *Lactobacillus fermentum*	[64]	Spain
	Yeasts	*Saccharomyces cerevisiae* (87–89%)	*Torulaspora delbrueckii*, *Hanseniaspora uvarum*/*guilliermondii*, *Cryptococus flavescens*, *Pichia anomala*, *Candida norvegica*		
Winery with GMP	Bacteria	*Micrococcus luteus* (67.4%), *Kytococcus sedentarius* (12.3%)	*Aerococcus*, *Brevibacillus*, *Bacillus*, *Corynebacterium*, *Leuconostoc*, *Lactococcus*, *Pediococcus*, *Staphylococcus*, *Streptococcus*	[86]	Malaysia
Five traditional and subterranean wine cellars (Tokaj wine district)	Molds Yeasts	*Penicillium*, *Rasamsonia*, *Talaromyces* and *Aspergillus* Yeasts not identified	*Alternaria*, *Aphanocladium*, *Aspergillus*, *Botrytis cinerea*, *Clasdosporium*, *Emericella nidulans*, *Epicoccum*, *Mucor*, *Scopulariopsis*, *Zasmidium cellare* *Candida membranifaciens*, *Debaryomyces subglobosus*	[73]	Hungary
One traditional wine cellar (in Sălacea), between April and May 2018	Molds	*Cladosporium*, *Penicillium*, *Aspergillus*,	*Trichoderma*, *Ulocladium*, *Geotrichum*, *Fusarium*, *Alternaria*	[87]	Romania

GMP—Good Manufacturing Practice; LAB—Lactic acid bacteria.

**Table 6:** Winery micro-organisms or molecules that can cause health issues for wine consumers, winery workers and visitors. Adapted from Benito [118]; Esposito et al. [119].

Metabolites/Micro-Organism	Taxa (Group/Genus/Species)	Health Issues
Biogenic amines (e.g., histamine, tyramine, putrescine, cadaverine)	LAB, some yeasts, *S. epidermis*, *E. faecium*	Allergenic, vasoactive, psychoactive effects
Aflatoxins, ochratoxin A	*Aspergillus* (*A. carbonarius*, *A. fumigatus*, *A. niger*, *A. tubingensis*, *A. japonicus, A. welwistichiae*), *Penicillium*	Carcinogenic, genotoxic, immunotoxic, hepatotoxic
Alternariol	*Alternaria* (*A. alternata*), *B. cinerea*	Mutagenic, carcinogenic
Fumonisins	*Fusarium* (*F. verticillioides*, *F. oxysporum*), *A. niger*, *A. welwitschiae*	Disrupt sphingolipid biosynthesis (inhibition of ceramide synthase)

LAB—Lactic acid bacteria (e.g., *Lactobacillus*, *Leuconostoc*, *Oenococcus*, *Pediococcus*).

## Data Availability

No new data were created or analyzed in this study.

## Supplementary Materials

The following supporting information can be downloaded at: https://www.mdpi.com/article/10.3390/microorganisms13030538/s1, Table S1: List of the bacteria and fungi (yeasts and molds) taxa reported in the winery environment (air, surfaces and must/wine). In bold: order level.
