# Supplementary material for: Microbiome and Microbiota Within Wineries: A Review
Source: Microorganisms. 2025 Feb 27;13(3):538. doi: 10.3390/microorganisms13030538 (PMC11944700; doi:10.3390/microorganisms13030538)
Supplement: Supplementary file 1 [file microorganisms-13-00538-s001.zip › microorganisms-3464752-supplementary.pdf]

Supplementary Information

Microbiome and Microbiota within Wineries: a review

Cristina Aires, Rita Maioto, António Inês, Albino Alves Dias, Paula Rodrigues, Conceição Egas, Ana Sampaio*

*Corresponding author asampaio@utad.pt

Centro de Investigação e Tecnologias Agroambientais e Biológicas (CITAB), Universidade de Trás-os-Montes a Alto Douro (UTAD), Quinta de Prados, 5000-801 Vila Real, Portugal

Table S1. List of the bacteria and fungi (yeasts and moulds) taxa reported in the winery environment (air, surfaces and must/wine). In bold, order level.

Taxa	Air	Surfaces	Must/wine
BACTERIA			
Acetetobacterales			
Acetobacteraceae		x	
Acetobacter sp.		x	
Gluconobacter sp.	x	x	
Gluconobacter cerinus			x
Gluconobacter oxidans			x
Komagataeibacter sp.	x		
Actinomycetales		x	
Alternomonadales		x	
Shewanella sp.		x	
Bacillales		x	
Bacillus sp.	x	x	
Bacillus pumillus	x		
Bacillus sphaericus	x		
Brevibacillus brevis	x		
Burkholderiales		x	
Telluria mixta			x
Caulobacterales		x	
Brevundimonas sp.		x	
Caryophanales			
Staphylococcus epidermidis	x		x
Staphylococcus saccharolyticus	x		x
Staphylococcus schleiferi	x		x
Enterobacterales		x	
Duffyella gerundensis			x
Enterobacter sp.	x		
Pantoea agglomerans			x

Enterobacteriaceae	x	x	
Escherichia coli			x
Flavobacteriales		x	
Chryseobacterium indologenes		x	
Flavobacterium sp.		x	
Hyphomicrobiales			
Methylobacterium sp.		x	x
Lactobacillales		x	
Aerococcus urinae	x		
Enterococcus sp.	x		
Enterococcus faecium	x		x
Lactobacillaceae		x	
Lactobacillus sp.	x		x
Lactobacillus brevis	x		
Lactobacillus casei	x		x
Lactobacillus casei/paracasei	x		
Lactobacillus fermentum	x		x
Lactobacillus mali	x		
Lactobacillus plantarum	x		x
Lactococcus lactis spp. *hordniae*	x		
Leuconostoc lactis	x		
Leuconostoc mesenteroides	x		x
Leuconostoc pseudomesenteroides	x		x
Lentilactobacillus hilgardii	x		
Oenococcus oeni	x	x	x
Pediococcus acidilactici	x		x
Pediococcus domnosus	x		x
Pediococcus parvulus	x		x
Pediococcus pentosaceus	x		x
Streptococcus porcinus	x		x
Micrococcales			
Micrococcus luteus	x		x
Micrococcus lylae	x		x
Kytococcus sedentarius	x		
Rothia dentocariosa	x		
Moraxellales			
Acinetobacter sp.			x
Pseudomonadales		x	
Pseudomonas sp.		x	x
Rhizobiales		x	
Rhodobacterales		x	
Rhodospirillales		x	

Sphingobacteriales		x	
Sphingomonadales		x	
Sphingomonas sp.		x	x
**FUNGI (moulds and yeasts)**			
**Phylum Ascomycota**			
**Botryosphaeriales**			
Columnosphaeria sp.			x
**Cladosporiales**			
Cladosporium sp.	x	x	x
Cladosporium cladosporioides	x	x	
Cladosporium herbarum	x		
Cladosporium macrocarpum	x		
Cladosporium ossifragi	x		
Cladosporium sphaerospermum	x		
**Chaetothyriales**			
Phialophora sp.	x		
Rhinocladiella atrovirens	x		
**Dothideales**			
Aureobasidium sp.	x	x	x
Aureobasidium pullulans	x	x	x
**Eurotiales**			
Aspergillaceae			x
Aspergillus sp.	x	x	
Aspergillus candidus	x		
Aspergillus clavatus	x		
Aspergillus conicus		x	
Aspergillus flavus	x		
Aspergillus fumigatus	x	x	
Aspergillus neoellipticus	x		
Aspergillus niger	x	x	x
Aspergillus nidulans		x	
Aspergillus ochraceus	x		
Aspergillus restrictus	x	x	
Aspergillus spelunceus	x	x	
Aspergillus tamari	x		
Aspergillus terreus	x		
Aspergillus varians	x	x	
Aspergillus versicolor	x		
Emericella sp.	x		
Emericella nidulans	x		
Eurotium sp.	x	x	

Eurotium amstelodami	x		
Eurotium chevalieri	x		
Eurotium herbariorum	x	x	
Eurotium intermedium	x		
Paecilomyces sp.	x	x	
Paecilomyces variotii	x		
Penicillium sp.	x	x	x
Penicillium adanetzioides	x		
Penicillium angulare	x		
Penicillium angulare	x		
Penicillium aurantiogriseum	x		
Penicillium brevicompactum	x		
Penicillium camemberti	x		
Penicillium canescens	x		
Penicillium carneum	x		
Penicillium chrysogenum	x		
Penicillium citreonigrum	x	x	
Penicillium citrinum		x	
Penicillium commune	x		
Penicillium corylophilum	x		
Penicillium crustosum	x		
Penicillium decumbens	x	x	
Penicillium dierckxii	x		
Penicillium echinulatum	x	x	
Penicillium expansum	x	x	
Penicillium glabrum	x	x	
Penicillium glandicola		x	
Penicillium griseofulvum	x		
Penicillium implicatum	x		
Penicillium italicum	x		
Penincillium janthinellum		x	
Penicillium montanense	x		
Penicillium nalgiovense			
Penicillium olsonii	x		
Penicillium oxalicum	x		
Penicillium paneum		x	
Penicillium pinophilum	x		
Penicillium purpurescens	x		
Penicillium restrictum	x		
Penicillium roqueforti	x	x	
Penicillium rugulosum	x		
Penicillium sclerotiorum	x		

Penicillium solitum	x	x	
Penicillium spinulosum	x	x	
Penicillium thomii	x	x	
Penicillium tricolor	x		
Penicillium variabile	x		
Penicillium verrucosum	x	x	
Penicillium viridicatum	x		
Penicillium waksmanii	x		
Rasamsonia brevistipitata	x	x	
Talaromyces diversus		x	
Talaromyces rugulosus		x	
Thysanophora penicillioides	x		
**Glomerellales**			
Verticillium sp.	x		
**Helotiales**			
Botrytis sp.	x	x	x
Botrytis cinerea	x	x	
Botrytis fabae	x		
Botryotinia sp.			x
Oidiodendron cereale	x		
Oidiodendron griseum	x		
**Hypocreales**			
Acremonium sp.	x		
Acremonium brachypenium		x	
Acremonium murorum	x		
Acremonium strictum	x		
Aphanocladium album	x		
Beauveria bassiana	x		
Fusarium sp.	x	x	
Fusarium oxysporum	x		
Fusarium poae	x		
Fusarium solani	x		
Geosmithia sp.	x		
Stachybotrys chartarum	x		
Stachybotrys microspora			x
Trichoderma sp.	x		
Trichoderma harzianum	x		
Trichoderma koningii	x		
Trichoderma viridae	x	x	
Trichothecium roseum	x		
**Incertae sedis**			
Arthrinium arundinis	x		

Arthrinium phaeospermum	x		
Arthrographis sp.	x		
Pyrenochaeta sp.		x	
**Microascales**			
Echinobotryum **sp.**	x		
Scopulariopsis sp.	x		
Scopulariopsis brevicaulis	x		
Scopulariopsis fusca	x		
Wardomyces inflatus	x		
**Mycosphaerellales**			
Mycosphaerella sp.	x		x
Zasmidium cellare	x	x	
**Pleosporales**			
Alternaria sp.	x	x	x
Alternaria alternata	x	x	
Alternaria porri		x	
Alternaria tenuissima		x	
Didymella sp.		x	
Epicoccum sp.	x		x
Epicoccum nigrum	x	x	
Neocucurbitaria sp.		x	
Phoma sp.	x		
Phoma glomerata	x		
Ulocladium sp.	x		
Ulocladium botrytis		x	
Ulocladium chartarum	x		
**Saccharomycetales**			
Arthroascus sp.	x		
Brettanomyces bruxellensis	x	x	x
Candida sp.		x	x
Candida boidinii	x		
Candida bombi			x
Candida burtonii	x		
Candida colliculosa			x
Candida intermedia	x	x	
Candida krusei			x
Candida membranifaciens	x	x	x
Candida norvegica	x		
Candida parapsilosis	x		
Candida pararugosa	x		
Candida quercitrusa	x		
Candida stellata			x

Candida sorbosa		x	
Candida tropicalis			x
Candida vinaria		x	
Candida vini			x
Candida wickerhamii		x	
Candida zemplinina		x	
Candida zeylanoides	x	x	
Dekkera sp.	x	x	x
Debaryomyces sp.		x	x
Debaryomyces hansenii	x	x	x
Debaryomyces subglobosus	x		
Geotrichum sp.	x		
Geotrichum candidum	x		
Hanseniaspora sp.		x	x
Hanseniaspora osmophila			x
Hanseniaspora uvarum			x
Hanseniaspora uvarum/ guillermondii	x		x
Issatchenkia sp.		x	x
Issatchenkia orientalis			x
Kloeckera apiculata	x	x	x
Lachancea sp.			x
Lachancea thermotolerans			x
Meyerozyma sp.		x	x
Metschinkowia sp.		x	x
Metschnikowia pulcherrima		x	x
Priceomyces sp.		x	
Pichia sp.	x	x	x
Pichia anomala	x	x	
Pichia farinosa	x		
Pichia fermentans	x		
Pichia kluyveri			x
Pichia kudriavzevii			x
Pichia membanifaciens	x	x	x
Saccharomyces sp.		x	x
Saccharomyces bayanus			x
Saccharomyces cerevisiae	x	x	x
Saccharomycodes sp.			x
Starmerella sp.		x	x
Torulaspora sp.			x
Torulaspora delbrueckii	x	x	
Wickerhamomyces sp.		x	x
Williopsis pratensis	x		

Zygoascus hellenicus			x
Zygosaccharomyces bailii		x	x
Zygosaccharomyces rouxii			x
Zygosaccharomyces veronae		x	x
**Schizosaccharomycetales**			
Schizosaccharomyces sp.			x
**Sordariales**			
Chrysonilia sp.	x		
Chrysonilia sitophila	x		
**Xylariales**			
Dicyma ampullifera	x		
**Phylum Basidiomycota**			
**Agaricales**			
Schizophyllum commune	x		
**Buckleyzymales**			
Buckleyzyma sp.		x	
**Filobasidiales**			
Filobasidium sp.		x	
Naganishia sp.		x	
**Moniliellales**			
Moniliella sp.	x		
Moniliella acetoabutens	x		
**Polyporales**			
Bjerkandera adusta		x	
Phanerochaete sp.	x		
Phanerochaete chrysosporium	x	x	
Trametes hirsuta	x		
Trametes versicolor	x		
**Russulales**			
Spiniger meineckellus	x		
**Tremellales**			
Bulleromyces sp.			x
Cryptococcus sp.	x	x	x
Cryptococcus albidus	x	x	
Cryptococcus carnescens	x		
Cryptococcus diffluens	x	x	x
Cryptococcus flavescens	x		
Cryptococcus infirmominiatum	x		
Cryptococcus laurentii	x	x	
Cryptococcus magnus	x		
Cryptococcus saitoi	x	x	
Cryptococcus uzbekistanensis	x		

Cryptococcus victoriae	x		
Vishniacozyma sp.		x	
**Sporidiobolales**			
Rhodotorula sp.	x	x	x
Rhodotorula diabovata			x
Rhodotorula glutinis	x	x	
Rhodotorula mucilaginosa	x		x
Rhodotorula slooffiae		x	
Sporobolomyces sp.	x	x	
Sporobolomyces roseus	x		
Sporidiobolus salmonicolor	x		
**Trichosporonales**			
Trichosporon sp.			x
**Wallemiales**			
Wallemia muriae		x	
Wallemia sebi	x	x	
**Phylum Mucoromycota**			
**Mortierellales**			
Mortierella sp.	x		
Mortierella alpina	x	x	x
**Mucorales**			
Absidia psychrophilia		x	
Mucor sp.	x	x	
Mucor circinelloides	x		
Mucor hiemalis	x		
Mucor plumbeus	x		
Mucor racemosus	x		
Rhizopus sp.	x	x	
Rhizopus microsporus	x		
Rhizopus oryzae	x		
Rhizopus stolonifer	x	x	
**Umbelopsidales**			
Umbelopsis isabellina	x	x	
